# Caveolin-mediated cytosolic delivery of spike nanoparticle enhances antitumor immunity of neoantigen vaccine for hepatocellular carcinoma

**DOI:** 10.7150/thno.85843

**Published:** 2023-07-16

**Authors:** Zhiwen Lin, Chenwei Jiang, Peiyuan Wang, Qingjing Chen, Bing Wang, Xinyue Fu, Yuzhi Liang, Da Zhang, Yongyi Zeng, Xiaolong Liu

**Affiliations:** 1 The United Innovation of Mengchao Hepatobiliary Technology Key Laboratory of Fujian Province, Mengchao Hepatobiliary Hospital of Fujian Medical University, Fuzhou 350025, P. R. China.; 2 Liver Disease Center, The First Affiliated Hospital of Fujian Medical University, Fuzhou 350005, People's Republic of China.; 3 CAS Key Laboratory of Design and Assembly of Functional Nanostructures, Fujian Institute of Research on the Structure of Matter, Chinese Academy of Sciences, Fuzhou 350002, P. R. China.; 4 Mengchao Med-X Center, Fuzhou University, Fuzhou 350116, P. R. China.; 5 Fujian Agriculture and Forestry University, Fuzhou 350002, P. R. China.

**Keywords:** Virus-like silicon nanoparticles, neoantigen, caveolin, immunotherapy, T cell immunoglobulin and mucin domain 3

## Abstract

**Rationale:** Although neoantigen-based cancer vaccines have shown promise in various solid tumors, limited immune responses and clinical outcomes have been reported in patients with advanced disease. Cytosolic transport of neoantigen and adjuvant is required for the activation of intracellular Toll-like receptors (TLRs) and cross-presentation to prime neoantigen-specific CD8^+^T cells but remains a significant challenge.

**Methods:** In this study, we aimed to develop a virus-like silicon vaccine (V-scVLPs) with a unique spike topological structure, capable of efficiently co-delivering a hepatocellular carcinoma (HCC)-specific neoantigen and a TLR9 agonist to dendritic cells (DCs) to induce a robust CD8^+^T cell response to prevent orthotopic tumor growth. We evaluated the antitumor efficacy of V-scVLPs by examining tumor growth and survival time in animal models, as well as analyzing tumor-infiltrating CD8^+^T cells and cytokine responses in the tumor microenvironment (TME). To evaluate the synergistic efficacy of V-scVLPs in combination with α-TIM-3 in HCC, we used an orthotopic HCC mouse model, a lung metastasis model, and a tumor rechallenge model after hepatectomy.

**Results:** We found that V-scVLPs can efficiently co-deliver the hepatocellular carcinoma (HCC)-specific neoantigen and the TLR9 agonist to DCs* via* caveolin-mediated endocytosis. This advanced delivery strategy results in efficient lymph node draining of V-scVLPs to activate lymphoid DC maturation for promoting robust CD8^+^T cells and central memory T cells responses, which effectively prevents orthotopic HCC tumor growth. However, in the established orthotopic liver tumor models, the inhibitory receptor of TIM-3 was significantly upregulated in tumor-infiltrating CD8^+^T cells after immunization with V-scVLPs. Blocking the TIM-3 signaling further restored the antitumor activity of V-scVLPs-induced CD8^+^T cells, reduced the proportion of regulatory T cells, and increased the levels of cytokines to alter the tumor microenvironment to efficiently suppress established orthotopic HCC tumor growth, and inhibit lung metastasis as well as recurrence after hepatectomy.

**Conclusion:** Overall, the developed novel spike nanoparticles with efficient neoantigen and adjuvant intracellular delivery capability holds great promise for future clinical translation to improve HCC immunotherapy.

## Introduction

Cancer vaccines (CVs) aim to boost tumor-specific T-cell responses with durable anticancer immune memory for selective tumor destruction and prevention of tumor recurrence 1-3. Neoantigens (NeoAgs), which arise from tumor-specific DNA alterations but are not present in normal cells, have emerged as promising targets for immunotherapy 4-9. NeoAgs with low central immune tolerance and high safety have been used in preclinical and clinical applications for solid tumors such as melanoma, glioma and hepatocellular carcinoma (HCC), *et al.* 10-17. However, in late-stage patients, the immune response and outcomes have been reported to be very limited 18. Recent evidences have shown that the anti-tumor activity of CVs is strongly associated with antigen-specific T cells. Dendritic cells (DCs) are crucial in initiating antigen-specific T cell responses by presenting NeoAgs to major histocompatibility complex upon maturation. Efficient lymph node (LN) drainage and cytosolic delivery of NeoAgs to DCs are prerequisites for priming antigen-specific T-cell responses 19-23. However, NeoAgs peptides always suffer from early enzymatic degradation, inefficient LN homing and inadequate cellular uptake by DCs, resulting in insufficient initiation of antigen-specific T-cell responses to fight against cancer 14,24,25.

Nanoparticle-mediated cytosolic delivery of antigens and adjuvants, along with effective lymph node drainage, has emerged as a promising strategy to promote DC activation, leading to the induction of potent antigen-specific T-cell responses 8,26-36. However, some cationic nanoparticle-based vaccines rely heavily on cationic compounds, which could potentially lead to unexpected toxicities such as cell shrinkage, reduced mitotic number and cytoplasmic vacuolization 37. Our and previous studies discovered a virus-like silicon particle (scVLP) with a unique spike topological structure similar to a virus. The spike structure can interact with the cell membrane, destabilize lipid membranes, and efficiently internalize into cells by mechanical action 38,39. Moreover, because scVLPs have a similar morphology to virus that can be efficiently internalized into lymphocyte cells 40, they are considered to be one of the most efficient and promising vectors for the development of cancer immunotherapy vaccines. In addition, dysfunctional CD8^+^T cells in the tumor microenvironment (TME) often upregulate inhibitory molecules including the T cell immunoglobulin and mucin domain (TIM-3) and programmed cell death protein 1 (PD-1) 41,42, to inhibit the magnitude of immune responses 43-48, while blocking such inhibitory signaling (such as TIM-3 signaling) is a promising strategy to rescue exhausted CD8^+^T cells for counter immunotherapy resistance 49-54.

**Scheme 1:**
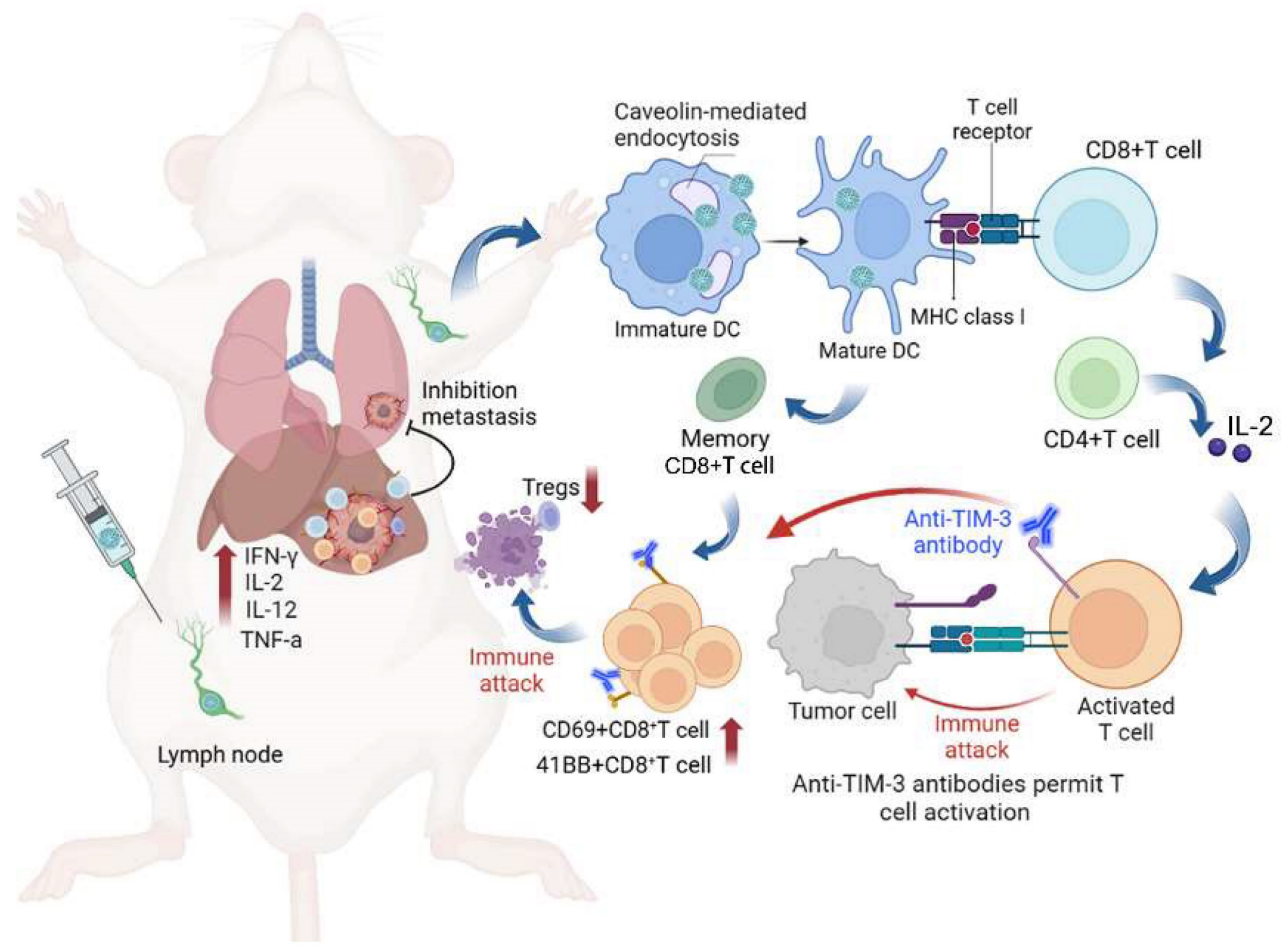
We herein developed a virus-like silicon nanovaccine (V-scVLPs) which could efficiently co-deliver the HCC-specific neoantigen and a TLR9 agonist to dendritic cells *via* caveolin-mediated endocytosis. This advanced delivery strategy results in robust CD8^+^T cell responses and central memory T cell responses for preventing orthotopic tumor growth. However, in the established tumor models, the inhibitory receptor of TIM-3 was significantly upregulated in CD8^+^T cells after immunization with V-scVLPs. Blocking the TIM-3 signaling further restored the antitumor activity of V-scVLPs-induced CD8^+^T cells, reduced the regulatory T cells, and increased cytokines to alter the tumor microenvironment for suppressing tumor growth, and inhibiting lung metastases as well as recurrence after hepatectomy.

Based on the above findings, the combination of scVLPs-based neoantigen nanovaccine (V-scVLPs) with anti-TIM-3 therapy would induce a robust T-cell response to enhance antitumor immunotherapy (**Scheme 1**). HCC, (highly lethal malignancy with immune tolerance and strong escape from immune surveillance) was used as a challenging tumor model 55-57. The results showed that V-scVLPs composed of Hepa1-6-specific NeoAgs and ODN-1826 induced the maturation of DCs for cross-presentation by activating TLR9 signaling. This activation was durable and resulted in the generating of antigen-specific CD8^+^T cells to prevent orthotopic HCC growth in a prophylactic setting. However, in a therapeutic setting, V-scVLPs-generated CD8^+^T cells were insufficient to completely inhibit tumor growth due to the upregulation of inhibitory molecules in CD8^+^ T cells. To overcome this obstacle, we combined V-scVLPs with TIM-3 blockade, which reduced the frequency of Tregs and restored the antitumor activity of CD8^+^ T cells. This combination therapy effectively controlled established tumor growth, prevented lung metastasis, and recurrence after hepatectomy.

## Materials and Methods

### Materials

3-aminopropyltrimethoxysilane (APTES), tetraethylorthosilicate (TEOS), anhydrous ethanol, acetone, cyclohexane, and Hoechst 33342 were obtained from Sigma. Hexadecyl trimethylammonium chloride (CTAB) and triethanolamine (TEA) were purchased from Sinopharm Chemical Reagent Co. Ltd. Dylight550-NHS was obtained from Thermo Fisher Scientific (USA). CCK8 assay kits were purchased from Dojindo Laboratories. Anti-CD11c-APC, anti-CD4-FITC, anti-CD86-PE-Cy7, anti-CD3-APC, anti-Foxp3-PE-Cy7, anti-IFN-γ-PE-Cy7, anti-CD80-PE, anti-CD8-PE, anti-TIM-3-FITC and anti-CD25-Percp-Cy5.5 were purchased from BioLegend, Inc. (San Diego, CA, USA). ELISA kits for the detection of IFN-γ, TNF-α, IL-2, IL-6 and IL-12p70 were purchased from Fankewei (Shanghai, China). Mouse liver cancer Hepa1-6 cell-specific NeoAgs (sequence: WDTCTTYKWQKTLEGHD and WDTCTTY KWQKTLEGHD-FAM) were synthesized by GenScript USA Inc. ODN-1826 (Cy3 labeled TCCATGACGTTCCTGACGTT or TCCATGACGTTCCTGACGTT) were synthesized by Sango Biotechnology Co., Ltd, China.

### Cell Culture and animals

Hepa1-6 cells and Hepa1-6-luc cells stably expressing luciferase (luc), were kept in DMEM (100 IU/mL penicillin-streptomycin and 10 % FBS (V/V)) at 37 °C. Male C57BL/6 mice (4-5 weeks) were obtained from China Wushi, Inc (Shanghai, China). Animals were treated in accordance with the Guide for the Care and Use of Laboratory Animals, and all animal procedures were approved by the Animal Ethics Committee of Mengchao Hepatobiliary Hospital of Fujian Medical University.

### Synthesis of V-scVLPs

To prepare the V-scVLPs vaccine, scVLPs were prepared using our previously reported method with slight modifications 38. We used a general two-phase method with 750 mg of CTAB dispersed in 60 mL deionized water, followed by adding 0.80 mL NaOH (0.1 M) as a reducing agent. The solution was then stirred for half an hour in a dimethyl silicone oil bath (60 °C); then, a mixture of 20 mL TEOS and cyclohexane (volume ratio was set to 1:4) was added to the aqueous layer. The whole system was kept in the oil bath for 72 h. The mesoporous silica was washed with DI water and ethanol (anhydrous) alternately for several times by centrifugation. Finally, the scVLPs were added in acetone (50 mL) and refluxed continuously (60 °C) overnight to remove the additional CTAB.

The resulting scVLPs were then mixed with 50 μL of APTES (ethanol) and refluxed under the 80 °C for 4 h through vigorous stirring. Then, the scVLPs-NH_2_ were purified through anhydrous ethanol, and dispersed in deionized water. Next, ^FAM^NeoAgs were added to different concentrations of scVLPs-NH_2_ and incubated for 6 h with shaking. After centrifugation, the supernatants were washed three times with Di-water, and the fluorescence intensity was recorded at 520 nm using a Spectra 206 Max M5. A standard curve was generated showing good linearity with ^FAM^NeoAgs concentrations from 0-20 µg/mL (Y = 43.79x - 15.687 R^2^ = 0.9996). Similarly, ^Cy3^ODN-1826 was added to various concentrations of scVLPs-NH_2_ solution with shaking for 6 h, and the linear fit of ODN-1826 from concentrations in the range of 0-2.5 µg/mL was Y = 238.22x - 23.613, R^2^ = 0.9955. The dose of ^FAM^NeoAgs and ^Cy3^ODN-1826 from the V-scVLPs was determined by measuring the UV-vis absorbance of the supernatants and comparing the readings to a corresponding standard curve. The dose of NeoAgs and ODN-1826 from V-scVLPs was 175.0 μg/mg and 20.0 μg/mg, respectively.

### Characterization

The morphology of both scVLPs and V-scVLPs was analyzed by transmission electron microscopy (TEM) using an FEI Tecnai G20 microscope (USA) and a scanning electron microscope (FEI Company, Hillsboro, OR). In addition, the particle size and zeta potential of both scVLPs and V-scVLPs were determined by using the Zetasizer NanoZS (ZEN3600, UK). The absorbance of these particles was recorded by using Spectro Max M5e (Germany). Finally, the Barrett-Joyner-Haenda (BJH) of scVLPs was performed on Zetasizer NanoZS (ZEN3600, UK).

### Subcellular localization and cellular internalization pathway of scVLPs in DC2.4 cells

To evaluate the subcellular localization of scVLPs, the DC2.4 cells were cultured on 35 mm glass-bottom Petri dishes at a density of 1 × 10^5^ per well and kept at 37 °C for 24 h. The cells were then stained with Lyso-Tracker Green and incubated with Cy5-NHS labeled scVLPs (^Cy5^scVLPs, 100 µg) for 3 h, 6 h and 12 h. respectively. After treatment, the cells were washed with PBS buffer (1 ×, pH 7.4) and imaged *via* confocal microscopy (CLSM) using an LSM 780 instrument from Germany. The nucleus was stained with Hoechst 33342 (excited at 405 nm), while the LysoTracker Green was excited by 488 nm and the Cy5 dye was excited at 633 nm. To explore the internalization pathways of ^Cy5^scVLPs, DC2.4 cells were again seeded onto 35 mm glass-bottom Petri dishes at a density of 1 × 10^5^ cells per well and cultured at 37 °C for 24 h. The cells were then preincubated with various endocytosis inhibitors (including chlorpromazine, 10 µg/mL, methy-β-cyclodextrin, 50 µM, or genistein, 200 µg/mL) for 30 min before being incubated with ^Cy5^scVLPs for 3 h at 37 °C. Finally, the cells were imaged by CLSM.

### Potential toxicity of V-scVLPs* in vitro* and *in vivo*

To evaluate the potential toxicity of V-scVLPs, the DC.2.4 cells were seeded in 96-well plates at a density of 1 × 10^4^ cells per well and kept for 24 h. Subsequently, fresh medium containing varying concentrations of V-scVLPs (ranging from 50 µg/mL to 1000 µg/mL) was added to the cells, which were then kept for 24 h. Cell viability was recorded by the CCK8 assay, as previously reported 38.

To evaluate the systemic toxicity of V-scVLPs, healthy mice were subcutaneously injected with V-scVLPs (at a dose of 400 µg) and sacrificed after either 3 or 14 days (with a sample size of n = 3 for each group). Main organs from the mice then collected and processed in 10% paraformaldehyde before sectioning and staining with hematoxylin and eosin (H&E). Finally, the samples were examined *via* microscopy.

### BMDCs Maturation and T cell activation

Maturation of BMDCs was analyzed according to our previously reported protocol 42. First, tibias and femurs were obtained from mice (4 to 5 weeks) and stored in medium on ice. Using scissors, the bone was trimmed, and the cells were subsequently flushed out with medium to remove any remaining cells. Debris was removed with a 40 µm cell strainer. The collected cells were then centrifuged and resuspended with the red blood cell lysis buffer to facilitate the lysis of red blood cells. Second, the collected cells were washed with medium and then centrifuged. The cells were then kept in a 6-well plate supplemented with medium and mouse granulocyte/macrophage colony stimulating factor (mGM-CFS, 20 ng/mL) and IL-4 (10 ng/mL) for 5 days. The inactivated marrow-derived dendritic cells were then kept in ODN-1826 (4 µg), NeoAgs (35 µg), Neo/ODN mixture (Neo/ODN vaccine, NeoAgs (35 µg) and ODN-1826 (4 µg), or V-scVLPs (scVLPs, 200 µg; NeoAgs, 35 µg and ODN-1826, 4 µg) for 48 h, respectively. The CD11c, CD80, and CD86 antibodies (eBioscience) were used to stain with the marrow-derived dendritic cells and evaluated through flow cytometry (FCM).

To assess T cell activation, the T cells were isolated from the spleen 41. Spleens were first harvested from mice (4-5 weeks) and stored in medium under ice. The obtained cells were subsequently washed out using medium, and any large debris was removed by using a 40 µm cell strainer. The obtained cells were then collected by centrifugation and resuspended in red blood cell lysis buffer for 10 minutes to split the cells. Following this, the treated cells were resuspended by medium and centrifuged once again. Next, the inactivated T cells were mixed with ODN-1826 (4 µg), NeoAgs (35 µg), Neo/ODN mixture (Neo/ODN, NeoAgs, 35 µg; ODN-1826, 4 µg), or V-scVLPs (scVLPs, 200 µg), NeoAgs (35 µg) and ODN-1826 (4 µg) activated BMDCs for 48 h of co-incubation. After treatment, the T cells were stained with 41BB, IFN-γ, CD69, CD3, and CD8 antibodies and analyzed *via* FCM.

### Draining LN homing of V-scVLPs

To evaluate the homing of V-scVLPs into LNs, C57BL/6 mice were subcutaneously injected with V-scVLPs (scVLPs, 400 µg; ^FAM^NeoAgs, 70 µg, ODN-1826, 8 µg) in a single groin. After 12 h, LNs were isolated from the mice and imaged using the IVIS®Spectrum *In vivo* Imaging System. The LNs were then abraded against a nylon filter and the harvested cells were stained with CD11c-APC for 30 minutes before being assessed by FCM to determine the number of FAM and APC positive staining cells. Additionally, the harvested cells were also stained with CD86-PE-Cy7, CD80-PE, and CD11c-APC antibodies for FCM analysis 8.

### Immunotherapeutic effect of V-scVLPs *in vivo*

To evaluate the immunotherapeutic effect of V-scVLPs, male C57BL/6 mice (4-5 weeks old) were purchased from China Wushi, Inc. (Shanghai, China). All animal procedures were performed in compliance with the “National Animal Management Regulations of China” and were approved by the Animal Ethics Committee of Mengchao Hepatobiliary Hospital of Fujian Medical University. For the experiment, Neo/ODN mixture (NeoAgs, 70 µg; ODN-1826, 8 µg) or V-scVLPs (scVLPs, 400 µg; NeoAgs, 70 µg; ODN-1826, 8 µg) were subcutaneously injected into the left groin of the mice every 4 days for a total of three times, respectively. The subcutaneous injection of PBS was used as the control. After injection of Hepa1-6-Luc cells (3 × 10^5^) directly into the liver lobe of the mice, bioluminescence images were captured using the UniNano imaging system and the bioluminescence signals were quantified as average radiance (photons, s^-1^cm^-2^sr^-1^) after luciferin injection (15 mg/mL, 200 µL per mouse).

### Antitumor immunotherapeutic effect of combination V-scVLPs with αTIM-3 *in vivo*

To assess the antitumor effect of the combined treatment, Hepa1-6-Luc cells (3 × 10^5^) were directly injected into the liver lobe. After 10 days, the established orthotopic Hepa1-6 liver cancer mice were confirmed by* in vivo* fluorescence imaging system and then randomly assigned to one of following four groups: PBS, αTIM-3, V-scVLPs or V-scVLPs combined with αTIM-3 (100 µg), respectively. In addition, mice were subcutaneously injected with PBS or V-scVLPs (scVLPs, 400 µg; NeoAgs, 70 µg; ODN-1826, 8 µg) on the day 10, 14 and 18 after inoculation with Hepa1-6-Luc cells, while αTIM-3 (100 µg per mouse) was intraperitoneally injected into mice on days 10, 14, and 18. Bioluminescence images were acquired every 7 days after intraperitoneal injection of luciferin (Perkin Elmer, 15 mg/mL, 200 µL per mouse), and bioluminescence signals were analyzed through the mean radiance (photons, s^-1^ cm^-2^ sr^-1^).

Assuming that two drugs, A and B, both have the ability to inhibit tumor growth, with drug A inhibiting Y_a_ percent of tumor growth at a dose a, and drug B inhibiting Y_b_ percent of tumor growth at a dose b, the combination of inhibition percent Y_ab,P_ can be estimated by the complete additivity principle of probability theory 58



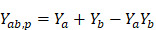



After calculating the combination of inhibition percentage Y_ab,P_, it is compared with the combination of inhibition proportion Y_ab,O_. In general, if Y_ab,O_ is greater than Y_ab,P_, the combined therapy is considered more effective than expected. Conversely, if Y_ab,O_ is less than Y_ab,P_, the combined therapy is less effective than expected. If Y_ab,O_ is equal to Y_ab,P_, the combined therapy is equivalent to the simple addition of two separate drugs. In summary, these scenarios can be summarized as follows.



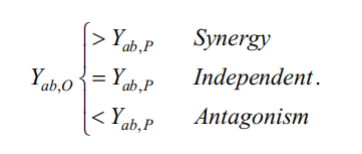



To analyze the percentage of lymphocytes in spleens or tumors after treatments, the tumors or spleens are excised from orthotopic HCC mice and the primary tumor is digested with enzymes such as 1 mg/mL of collagenase type IV, 0.2 mg/mL of hyaluronidase, 0.02 mg/mL of DNase I. The resulting single-cell suspension is then colored with various antibodies to analyze the infiltrated T cells and Tregs. Specifically, anti-CD4-FITC (eBioscience™, 11-0042-85), anti-CD8-PE (eBioscience™, 12-0081-82), anti-CD3-APC (eBioscience™, 17-0032-82), anti-CD62L-PerCP-Cy5.5 or anti-CD44-PE-Cy7antibodies were used for T cell analysis. For Treg infiltration analysis, the cell suspension was colored with anti-CD4-FITC, anti-CD25-per-Cy5.5 (eBioscience™ 45-0251-82), and anti-Foxp3-PE-Cy7 (eBioscience™, 25-5773-8 2) antibodies.

To further evaluate the pathological changes of the tumors after treatment, the tumor sections were stained for H&E analysis. Meanwhile, to analyze the secreted cytokines in the tumors, the treated tumors were harvested and lysed in RIPA lysis solution containing a 1 × Stop protease inhibitor cocktail, homogenized by magnetic beads (60 Hz) for 6 min and then centrifuged at 4 °C. The resulting supernatants were then analyzed by using ELISA kits to detect the presence of TNF-α, IFN-γ, IL-6, IL-2, and IL-12 in accordance with the manufacturer's statements.

To evaluate the tumor recurrence prevention ability of the combination treatment of V-scVLPs (scVLPs, 400 µg; NeoAgs, 70 µg; ODN-1826, 8 µg) and αTIM-3 (100 µg per mouse), Hepa1-6-Luc cells (3 × 10^5^) mixed with Matrigel suspension (25 μL) were directly injected into the liver lobe of mice. After 10 days, the established orthotopic Hepa1-6 liver cancer mice were confirmed by UniNano *in vivo* imaging system through luciferin injection, and then the αTIM-3 combined with V-scVLPs was injected intraperitoneally into the mice on the day 7, 11, and 15 after inoculation of Hepa1-6-Luc cells, the tumor and the liver lobe where the tumor is located were removed together on 17^th^ day. The same batch of mice, as well as mice after surgery, were again inoculated with Hepa1-6-Luc cells (3 × 10^5^), and bioluminescence images were collected after injection of luciferin (15 mg/mL, 200 µL per mouse). Bioluminescence signals were the quantified as mean radiance (photons, s^-1^cm^-2^sr^-1^).

### Statistical analysis

Statistical analysis of all data was performed using one-way analysis of variance (ANOVA) for comparisons between multiple groups. While two-tailed Student's t-test was used for comparisons between two groups. GraphPad Prism 8.0 software was used to perform these analyses. Survival curves were constructed by using Kaplan-Meier estimates and tested using the log-rank test. The significance level was set at *p < 0.05 for statistical significance. **p < 0.01, ***p < 0.001, ****p < 0.0001. Pearson's correlation coefficient was used to evaluate the correlation matrices. All the data are presented as means ± standard (n ≥ 3). Data are expressed as mean ± SD.

## Results and Discussion

### Characterization of V-scVLPs

**Figure 1:**
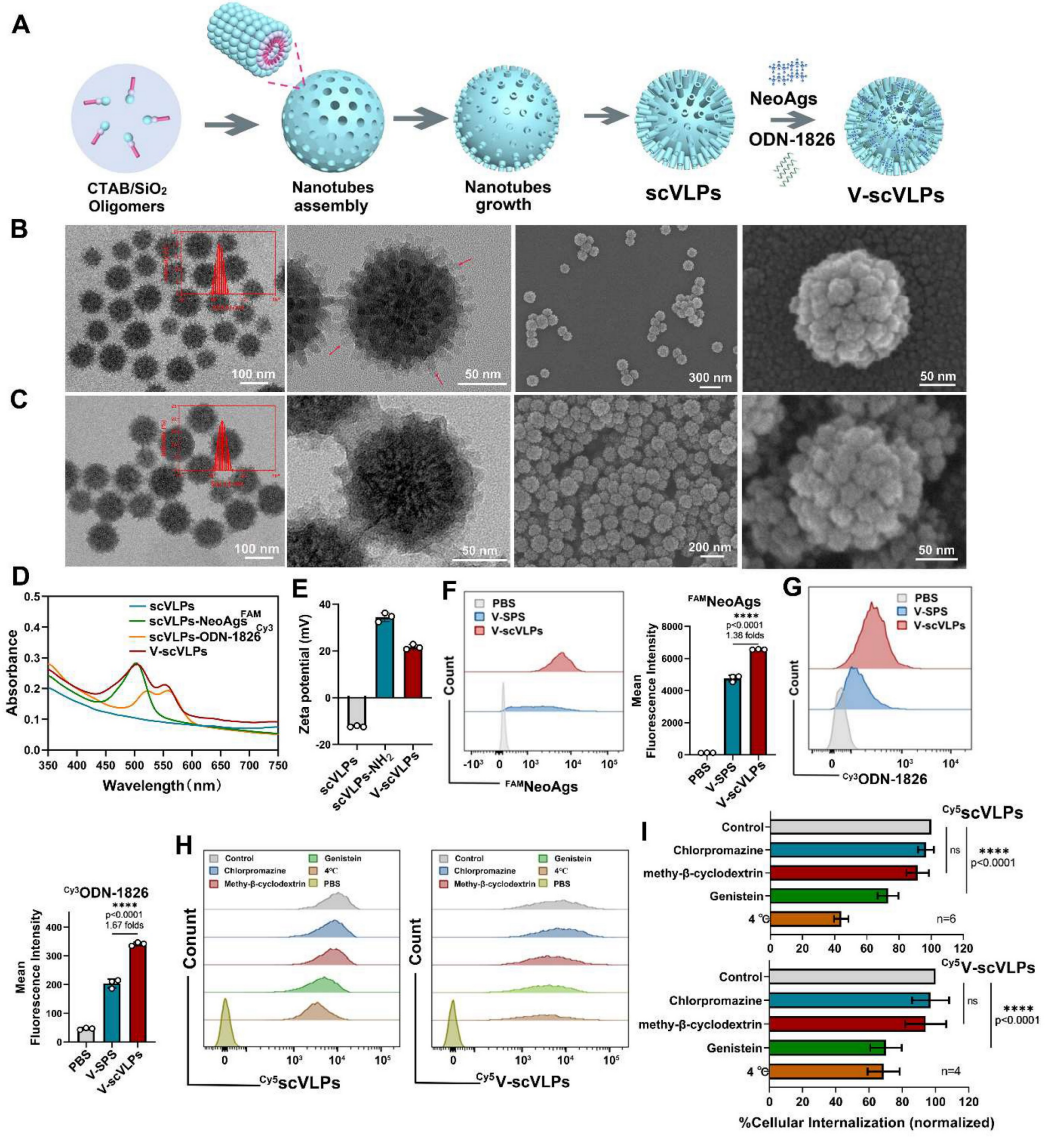
Characteristic of V-scVLPs. (A) Schematic representation of the preparation of V-scVLPs. The TEM and SEM image of scVLPs (B) and (C) V-scVLPs. The size distribution of scVLPs and V-scVLPs was shown in the insert picture. (D) The UV-vis absorbance of scVLPs, scVLPs-NeoAgs^FAM^, scVLPs-ODN-1826^Cy3^ and V-scVLPs (NeoAgs^FAM^ and ODN-1826^Cy3^). (E) Zeta potential of scVLPs, scVLPs-NH_2_ and V-scVLPs, (n = 3). (F) DC2.4 cell uptake and (G) FCM analysis of^ FAM^NeoAgs and ^Cy3^ODN-1826 from smooth MSN (SPS)-based nanovaccine (V-SPS) and V-scVLPs by FCM analysis, (n = 3). (H) FCM analysis and (I) cellular internalization of ^Cy5^scVLPs or V-^ Cy5^scVLPs-treated DC2.4 cells with pre-treatment of different endocytosis inhibitors, including the genistein, chlorpromazline, methy-beta-cyclodextrin and PBS buffer, or at 4^o^C. Positive controls show scVLPs treated DC2.4 cells without any endocytosis inhibitor treatment. Statistical analysis was performed with ANOVA analysis, ****p < 0.0001, (n = 3). Data are expressed as mean ± SD.

The uniform scVLP with an inner nanoparticle surrounded by epitaxial perpendicular mesopore nanotubes was prepared by our previously reported methods 38 (**Figure 1A**). Transmission electron microscopy (TEM) images revealed the spiky structure of scVLPs on silica, with an average length of 17 nm, and the scVLPs had an average particle diameter of 122.8 ± 6.0 nm, with hydrodynamic size of 159.0 ± 2.0 nm (Figure 1B, insert). TEM and scanning electron microscopy (SEM) revealed the typical virus-like morphology of scVLPs. Barrett-Joyner-Haenda (BJH) desorption revealed two pore sizes of 1.7 nm and 30 nm, respectively, which corresponded to the pore size of the spiky nanostructure and the interspace size of scVLPs (Figure S1). Next, scVLPs were mixed with NeoAgs and ODN-1826 for 6h, and purified by centrifugation. TEM and SEM images revealed V-scVLPs with an average particle diameter of 132.3 ± 6.4 nm, and the hydrodynamic size increased up to 187.0 ± 3.0 nm (Figure 1C). In addition, we compared the loading efficiency of NeoAgs or ODN-1826 in scVLPs or smooth mesoporous silica (SPS) by calculating the absorbance values of ^FAM^NeoAgs at 520 nm and ^Cy3^ODN-1826 at 570 nm using standard curves, respectively (Figure S2-3). SPS with similar particle size distribution was confirmed by TEM (Figure S4). As shown in Table S1 and Figure S5, the scVLPs allowed the loading of NeoAgs at 459 μg/mg (31.46 wt% *vs* 23.98 wt% in SPS) and the loading of ODN-1826 at 47.88 μg/mg (4.57 wt% *vs* 2.37 wt% in SPS), indicating the efficient loading capability of scVLPs. In addition, the UV-vis spectra of V-scVLPs showed two characteristic peaks at 495 and 550 nm, which consisted of the peak of ^FAM^NeoAgs and ^Cy3^ODN-1826 in the scVLPs, respectively, indicating the successful co-loading of NeoAgs and ODN-1826 in the scVLPs (Figure 1D). The zeta potential of V-scVLPs decreased from +34.47 ± 1.92eV (scVLPs) to +21.67 ± 1.12eV due to the negative charge of NeoAgs and ODN-1826 (Figure 1E), which may benefit cellular uptake by APCs.

### Cellular uptake mechanism and localization of V-scVLPs

Efficient cytosolic delivery of antigens and adjuvants to DCs enables them to cross-present and generate a potent antigen-specific T-cell immune response 26-31,59. The unique topological features of V-scVLPs prompted us to evaluate their cytosolic delivery efficiency *in vitro*. Using equal amounts of NeoAgs and ODN-1826 loaded into SPS as the control, we quantified them by FCM. As shown in Figure 1F-G, the mean fluorescence intensity (MFI) of NeoAgs (FAM) and ODN-1826 (Cy3) in V-scVLPs treated DC.2.4 cells were 1.38-fold and 1.67-fold higher, respectively, compared to SPS-based vaccine treated cells. This indicated a highly efficient intracellular delivery of NeoAgs and ODN-1826 by scVLPs. To investigate the cellular uptake pathway of V-scVLPs, we used various endocytosis inhibitors including the chlorpromazine (CMZ, clathrin-mediated endocytosis inhibitor), methy-β-cyclodextrin (Mβ-CD, a lipid raft-mediated endocytosis inhibitor) or genistein (a caveolae-mediated endocytosis inhibitor) 38,61. We treated DC.2.4 cells with these inhibitors to block the uptake for 30 min. After blocking, we observed that the MFI of Cy5-tagged V-scVLPs (V-^Cy5^scVLPs) in DC.2.4 cells did not significantly decrease after treatment with CMZ compared to cells treated with V-^Cy5^scVLPs alone (control), and similar results were obtained from Mβ-CD treatment (Figure 1H-I). However, the MFI of V-^Cy5^scVLPs-treated cells decreased significantly after genistein treatment or when maintained at 4^o^C. These results suggest that the caveolae-dependent endocytosis mechanism is responsible for the uptake of V-scVLPs by DCs.2.4. Furthermore, we found that the endocytosis mechanism for scVLPs was similar to that of the V-scVLPs.

**Figure 2:**
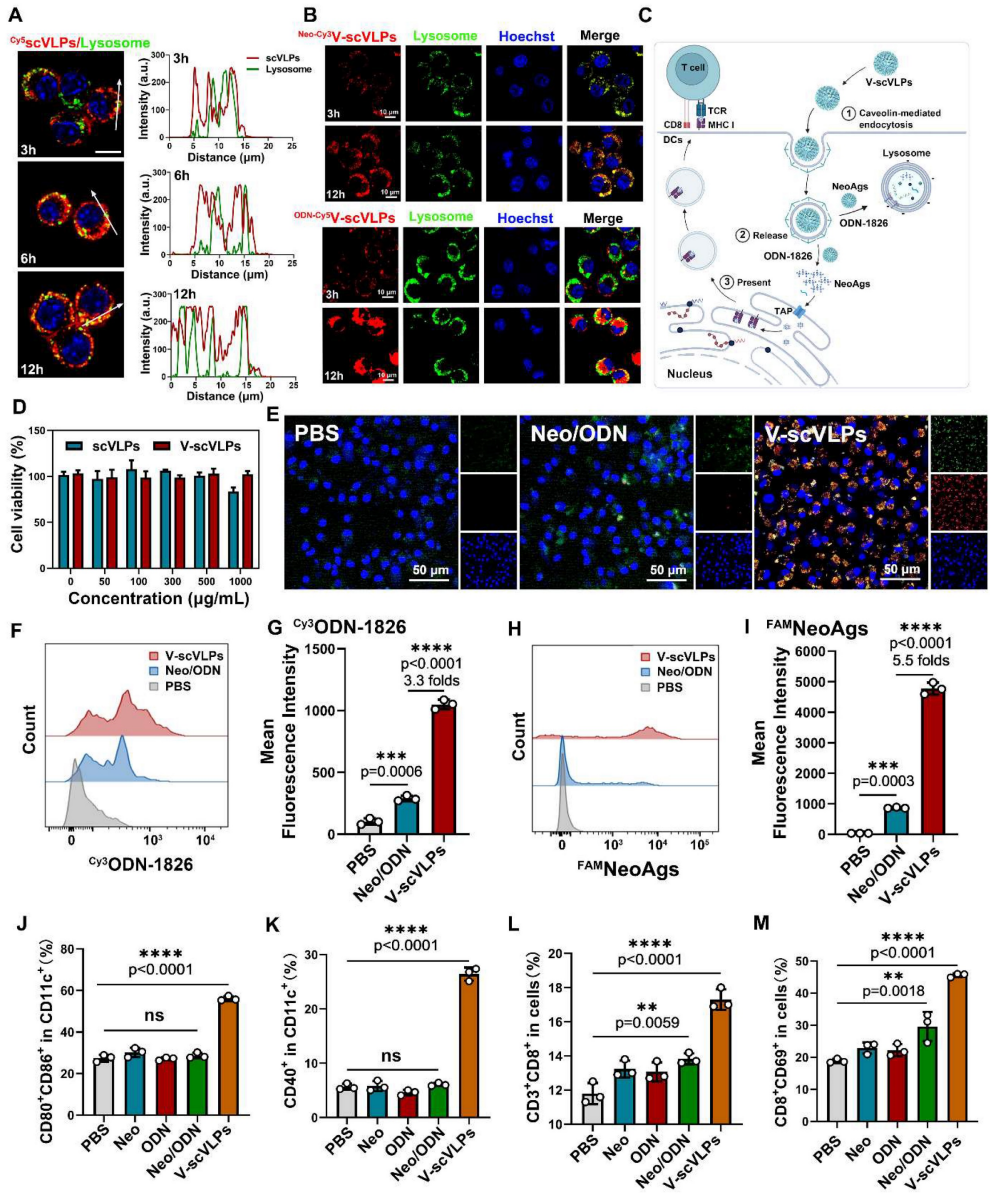
Intracellular uptake and activation of DCs and T cells by V-scVLPs. (A) The CLSM images of DC2.4 cells treated with Cy5-labeled scVLPs (red) at different time points (3, 6 or 12 h). Nuclei were stained with Hoechst (blue). Lysosome (green) was stained with LysoTracter Green. Scale bars: 10 μm. Spatial-intensity profiles of DC2.4 cells along the white arrows in panels. (B) The sub-localization of V-scVLPs (load ^Cy3^NeoAgs/ODN-1826) or V-scVLPs (load NeoAgs/ ^Cy5^ODN-1826) and incubated them with DCs.2.4 cells. Nuclei were stained with Hoechst (blue). Lysosome (green) was stained with LysoTracter Green. Scale bars: 10 μm. (C) Schematic illustration of the internalization and localization of V-scVLPs in BMDC cells. (D) Cell viability of bone marrow DCs (BMDCs) from C57bL/6 mice (4-5 weeks) after treatment with different concentration of scVLPs or V-scVLPs for 24h, (n = 7). (E) The cellular uptake of Neo/ODN vaccine or V-scVLPs by BMDCs after 12h co-incubation. BMDCs treated with PBS buffer was used as control. The red fluorescence was from Cy3 labeled ODN-1826, and the green fluorescence was from FAM labeled NeoAgs. (F) and (G) The FCM analysis and mean fluorescence intensity of ^Cy3^ODN-1826 from V-scVLPs treated BMDCs. (H) and (I) The FCM analysis and mean fluorescence intensity of ^FAM^ NeoAgs from V-scVLPs treated BMDCs, (n = 3). (J) The activated BMDCs and the maturation of BMDCs (K) after co-incubation with PBS, ODN-1826, NeoAgs, Neo/ODN vaccine or V-scVLPs for 48 h and calculated by FCM with using CD40, CD80 and CD86 antibody staining, respectively, n = 3. Data are expressed as mean ± SD. (L) The percentage of CD8^+^CD3^+^T cells and (M) The activation of CD69^+^CD8^+^T cells by PBS-, ODN-1826-, NeoAgs-, Neo/ODN vaccine- or V-scVLPs-treated DCs after 72 h incubation, and calculated by FCM, (n = 3). Statistical analysis was performed with ANOVA analysis, *p<0.05, **p<0.01, ***p<0.001, ****p<0.0001. Data are expressed as mean ± SD.

To visualize the subcellular localization of scVLPs in DC.2.4 cells, confocal microscopy (CLSM) imaging was performed. The CLSM imaging showed that our ^Cy5^scVLPs (red) were predominantly localized in the cytoplasm within the first 3h (Figure 2A). Meanwhile, a slight overlap between lysosome (green) and ^Cy5^scVLPs (red) was detected, with Pearson's correlation coefficients (PCCs) of 0.33 ± 0.02 (below the threshold of > 0.5) 62 (Figure S6). After prolonging the co-incubation time for 12 h, the overlapping signal was significantly increased with PCCs reaching 0.65 ± 0.06. This finding suggests that a fraction of scVLPs actively translocate to lysosomes through electrostatic interactions. To further investigate the sublocalization of NeoAgs and ODN-1826 from V-scVLPs, we loaded ^Cy3^NeoAgs/ ODN-1826 or NeoAgs/^Cy5^ODN-1826 into scVLPs and incubated them with DCs.2.4 cells (Figure 2B and 2C). The results showed that part of ^Cy3^NeoAgs (red) in V-scVLPs were located in the cytoplasm after the initial 3 h incubation period, and part of them were also detected in the lysosome. Similar results were observed for ^Cy5^ODN-1826 (red) from V-scVLPs treated DCs2.4 cells. After prolonging the co-incubation time for 12 h, certain amount of ^Cy3^NeoAgs appeared in the lysosomes, while also a large amount of ^Cy5^ODN-1826 appeared in the cytoplasm. This may reduce the unnecessary degradation of ODN-1826 in the lysosome and facilitate the sustained activation of TLR9 signaling to enhance the cross-presentation of NeoAgs by DCs.

### Efficient Activation of DCs by V-scVLPs to elicit activated CD8^+^T cells *in vitro*

To evaluate the activation of DCs by V-scVLPs, we first assessed the potential toxicity of V-scVLPs on bone marrow-derived DCs (BMDCs) using CCK8 (Figure 2D). After co-incubation for 24 h, the viability of V-scVLPs-treated BMDCs remained above 98.7% even at a concentration up to 1000 μg/mL. In contrast, scVLPs showed slight toxicity to BMDCs, with only 82.2% viable cells, due to the strong positive charge of scVLPs. Next, we compared the efficiency of V-scVLPs and Neo/ODN mixture for cellular uptake and activation of BMDCs. CLSM images showed significantly higher fluorescence of ^FAM^NeoAgs and ^Cy3^ODN-1826 in V-scVLPs treated BMDCs (Figure 2E). FCM results showed that the MFI of^ FAM^NeoAgs or ^Cy3^ODN-1826 from V-scVLPs treated BMDCs cells was 5.5-fold and 3.6-fold higher than in Neo/ODN mixture treated cells (Figure 2F-I), indicating efficient delivery of NeoAgs and adjuvant to BMDCs by scVLPs.

Unmethylated CpG-ODN can bind to Toll-like receptor 9 (TLR9) and activate nuclear factor kappa-B (NF-kB), leading to the induction of cytokine genes in DCs 60. We investigated whether V-scVLPs also could upregulate NF-kB in BDMCs by Western blot. After co-incubation for 48 h, we observed an upregulation of NF-kB in BDMC cells treated with V-scVLPs compared to the control, indicating the activation of the TLR9 signaling pathway (Figure S7). Next, the expression of CD40, CD80 and CD86 in CD11c^+^ BMDCs was assessed by FCM. The CD80 and CD86 co-stimulatory molecules were also significantly up-regulated in CD11c^+^ BMDCs treated with V-scVLPs (56.10% ± 1.14%), which was much higher than those treated with NeoAgs alone (30.20% ± 2.26%), ODN-1826 alone (27.10% ± 0.72%), or BMDCs treated with Neo/ODN-mixture (28.63% ± 1.37%) (Figure 2J and S8). In addition, FCM results showed that V-scVLPs more efficiently activated the maturation of BMDCs by up-regulating their co-stimulatory molecule CD40 (26.43% ± 1.19%), which was significantly higher than that of NeoAgs-treated cells alone (5.81% ± 0.93%), ODN-1826-treated cells alone or Neo/ODN mixture -treated cells (6.07% ± 0.30%) (Figure 2K and S9). These data demonstrated the efficient activation of BMDCs by V-scVLPs, which could promote T-cell activation. To test this hypothesis, V-scVLPs-matured BMDCs were co-cultured with naive T cells and analyzed by FCM. The results showed that V-scVLPs-activated BMDCs efficiently increased the proportion of CD8^+^CD3^+^T cells (17.30% ± 0.61%) compared to NeoAgs-treated (13.23% ± 0.49%), ODN-1826 treated (13.07% ± 0.55%) or Neo/ODN mixture-treated (13.83% ± 0.32%) groups (Figure 2L and S10). Additionally, these matured DCs induced by V-scVLPs activated a high percentage of CD69^+^CD8^+^T cells (45.60% ± 0.61%), which was much higher than that of NeoAgs treatment alone (22.90% ± 1.73%), ODN-1826 treatment alone (22.20% ± 1.90%), or Neo/ODN mixture treatment (29.60% ± 4.54%), respectively (Figure 2M and S11). These results demonstrate that V-scVLPs were superior in enhancing cellular uptake, activating DCs and stimulating navie T-cell activation.

### Efficient lymph node homing and immunoprophylaxis effect of V-scVLPs

**Figure 3:**
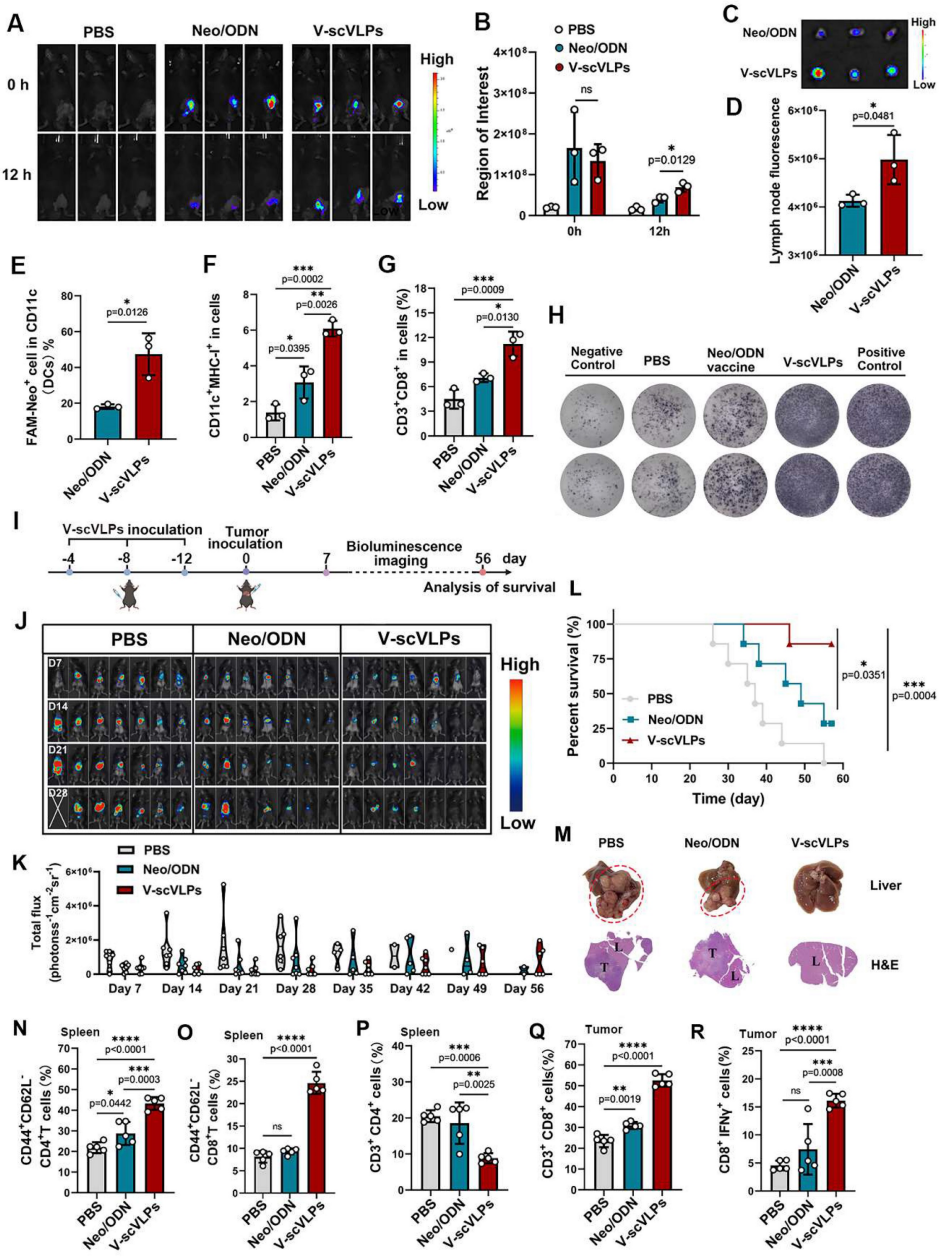
Immunoprophylaxis effect of V-scVLPs. (A) and (B) *In vivo* fluorescence images and intensity of ^FAM^V-scVLPs or ^FAM^Neo/ODN mixture treated C57BL/6 mice after injection for 0, 12 h, respectively. (C) and (D) *Ex vivo* fluorescence images and intensity of ^FAM^NeoAgs in LNs that were isolated from V-scVLPs or Neo/ODN mixture treated mice. (E) The CD11c^+^BMDCs uptake of ^FAM^NeoAgs from V-scVLPs or Neo/ODN that were isolated from LNs (n = 3). (F) The over-expression of MHC-I in CD11c^+^DCs in LNs after V-scVLPs or Neo/ODN vaccine treatment of C57BL/6 mice, (n = 3). (G) The percentage of CD3^+^CD8^+^T cells in spleen after V-scVLPs or Neo/ODN treatment of C57BL/6 mice, (n = 3). (H) ELISPOT assay analysis of IFN-γ spot-formation of PBMCs that isolated from C57BL/6 mice after s.c. injection of PBS, V-scVLPs or Neo/ODN vaccine for 14 days, and then co-incubated with NeoAgs for 48 h. (I) Schematic illustration of the process of *s.c.* injection of V-scVLPs in C57bL/6 mice for three times every 4 days and then intrahepatic inoculation of Hepa1-6-luc tumor cells to evaluate the antitumor effect of V-scVLPs. (J) Bioluminescence imaging and (K) total flux intensity of orthotopic Hepa1-6-luc bearing mice and total flux at 0, 7, 14, 21 days before and after receiving different treatments as indicated (n = 7). (L) The survival curve of orthotopic Hepa1-6-luc bearing mice after indicated treatment (n = 7). (M) Photomicrograph and H&E staining of liver and orthotopic tumor from one of the mice treated with PBS, V-scVLPs or Neo/ODN vaccine treatment, respectively. (N) and (O) The percentage of effector memory CD4^+^ and CD8^+^T cells (T_EM_) (CD44^+^CD62^-^T cells) in the spleen after receiving PBS, V-scVLPs or Neo/ODN vaccine treatment, respectively, (n = 5). (P) The CD3^+^CD4^+^T cells and (Q) the CD3^+^CD8^+^T cells in the tumors after receiving PBS, V-scVLPs or Neo/ODN vaccine treatment, respectively, (n = 5). (R) Percentage of IFN-γ^+^CD8^+^T cells in the tumors after receiving different treatment as indicated (n = 5). Statistical analysis was performed with ANOVA analysis, *p<0.05, **p<0.01, ***p<0.001, ****p<0.0001. Data are expressed as mean ± SD.

Next, to evaluate the lymph node homing effect of V-scVLPs administered by subcutaneous (s.c.) injection, *in vivo* NIR fluorescence imaging was performed (Figure 3A). As shown in Figure 3B-D, we observed a higher fluorescence intensity of^ FAM^NeoAgs from V-scVLPs retained in the groin and draining lymph nodes (1.2-fold) compared to mice treated with Neo/ODN mixture after 12 h. Additionally, we evaluated the uptake of V-scVLPs by CD11c^+^DCs, and the proportion of FAM^+^CD11c^+^DCs (47.40% ± 11.72%) in V-scVLPs treated mice was much higher than in ^FAM^Neo/ODN vaccine-treated mice (18.06% ± 1.32%), indicating the efficient uptake of V-scVLPs by lymphoid DCs* in vivo* (Figure 3E and S12). In addition, the lymphoid DC maturation was analyzed by staining with CD80, CD86 and CD11c antibodies through FCM, and the results showed that the co-expression of CD80^+^CD86^+^ in 58.07% ± 5.18% lymphoid DCs was found in the V-scVLPs treated group, which was higher than that of Neo/ODN treated mice (44.73% ± 2.25%) (Figure S13).

Further studies showed that the efficient activation of lymphoid DCs by V-scVLPs could subsequently enhance the expression of MHC-I in CD11c^+^DCs, which could be more beneficial for the activation of CD8^+^T cells (Figure 3F and S14). Therefore, we assessed the proportion of CD8^+^T cells in the spleen. A relatively high population of CD8^+^CD3^+^T cells (11.21% ± 1.50%) was observed in the spleen of V-scVLPs-treated mice, compared to the Neo/ODN-mixture treated group (7.08% ± 0.52%) (Figure 3G and S15). The enzyme-linked immunospot assay (ELISPOT) showed the significantly higher IFN-γ secretion in V-scVLPs treated mice compared to the Neo/ODN mixture treated group, indicating a strong T cell immune response after immunization with V-scVLPs* in vivo* (Figure 3H).

Next, the prophylactic effect was evaluated after immunization with V-scVLPs for three times every 4 days (Figure 3I). Subsequently, Hepa1-6-luc cells were injected into the liver. As shown in Figure 3J-K, the mice treated with V-scVLPs showed significantly reduced tumor luciferase activity, with 4/7 mice being completely cured without any trace of tumor at day 28, compared to the Neo/ODN mixture (2/7) or PBS-treated groups (0/7). After prolonging the observation for 56 days, the 3/7 mice that were cured in the V-scVLPs treated groups still showed no tumors, compared to PBS (0/7) or Neo/ODN mixture (0/7) treated groups (Figure 3L and S16). In addition, immunization with V-scVLPs significantly extended the survival of 6/7 mice beyond 56 days compared to the PBS treated group where none of the mice survived beyond 56 days. Only 2/7 mice treated with Neo/ODN mixture survived beyond 56 days. The excellent immunoprophylactic effect of V-scVLPs was further confirmed by photographing the *ex*-liver tissues with tumors or by H&E analysis (Figure 3M). In addition, V-scVLPs were found to induce a strong immune memory with a higher expression of co-stimulatory receptor (CD44^+^CD62L^-^) in CD4^+^T cells (43.24% ± 3.09%) and in CD8^+^T cells (23.97% ± 1.34%), compared to Neo/ODN mixture treated mice (in CD4^+^ T cells, 28.74 ± 5.60%; in CD8^+^ T cells, 9.62 ± 0.4%), indicating enhanced activation of T_EM_ cell by V-scVLPs (Figure 3N-O and S17-18). Furthermore, mice-treated with V-scVLPs showed a lower CD3^+^CD4^+^T cell population compared to other treatment groups (Figure 3P and S19). While a higher proportion of CD3^+^CD8^+^T cells (52.56% ± 3.01%) and IFNγ^+^CD8^+^T cells (16.12% ± 1.22%) was observed in V-scVLPs treated mice, compared to Neo/ODN mixture treated mice (CD8^+^T cells of 30.94% ± 1.67% and IFNγ^+^CD8^+^T cells of 7.45% ± 4.50%), which could effectively prevent orthotopic HCC growth (Figure 3P-R and S20-21). These findings suggest that V-scVLPs are potent in activating CD8^+^T cells to enhance the neoantigen therapies.

### V-scVLPs suppress tumor growth and prolong survival in a therapeutic setting by combined with anti-TIM3 therapy

**Figure 4:**
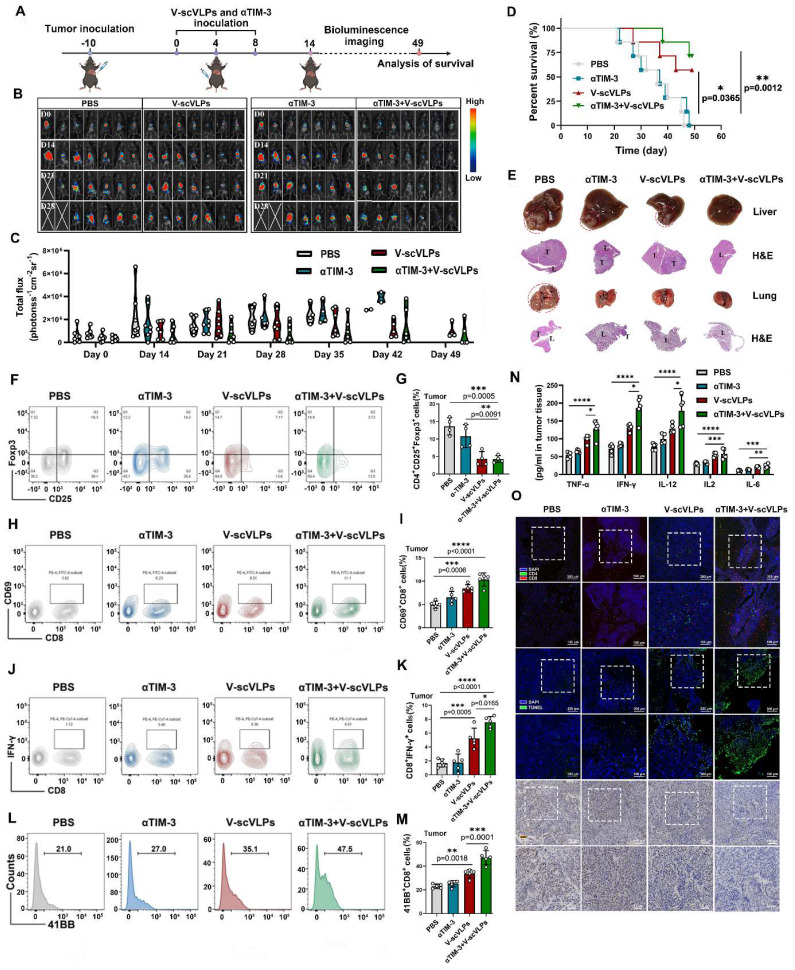
Synergistic antitumor effect of V-scVLPs and anti-TIM3 therapy. (A) Schematic illustration of the delivery process of V-scVLPs and anti-TIM3 antibody in established orthotopic Hepa1-6-luc tumor models. (B) Bioluminescence imaging and (C) total flux intensity of established orthotopic Hepa1-6-luc bearing mice and total flux at 0, 14, 21 and 28 days before and after receiving different treatments as indicated. (n = 7). (D) Photomicrograph and H&E staining of liver and orthotopic tumor from one of the mice that received PBS, V-scVLPs or Neo/ODN treatment, respectively. (E) The survival curve of orthotopic Hepa1-6-luc bearing mice after the indicated treatment (n = 7). (F) and (G) The percentage of Foxp3^+^CD25^+^CD4^+^T cells in orthotopic tumors after receiving different treatment as indicated, n = 4. (H) and (I) Percentage of CD69^+^ CD8^+^T cells in the tumors after receiving different treatment as indicated (n = 5). (J) and (K) Percentage of IFN-γ^+^CD8^+^T cells in the tumors after receiving different treatment as indicated (n = 5). (L) and (M) Percentage of 41BB^+^CD8^+^T cells in the tumors after receiving different treatment as indicated (n = 5). (N) Cytokine levels of IFN-γ, IL-2, IL-12, IL-6 and TNF-α in tumors isolated from differently treated mice by ELISA analysis. (O) CLSM image of CD4^+^T cells (green) and CD8^+^T cells (red) in tumors at day 14 after different treatments as indicated. scale bar, 100 μm. In addition, the TUNEL staining (green fluorescence) and Ki67 staining of tumor sections after receiving different treatments were imaged by CLSM as indicated. Statistical analysis was performed with ANOVA analysis, *p<0.05, **p<0.01, ***p<0.001, ****p<0.0001. Data are expressed as mean ± SD.

Encouraged by the excellent tumor control effect of V-scVLPs in a prophylactic setting, we investigated whether these V-scVLPs could control established orthotopic HCC growth (Figure 4A). As shown in Figure 4B, 4C and S22, although V-scVLPs-treated mice showed reduced tumor luciferase activity in 3/7 mice, this treatment did not completely control tumor growth at either day 21 or 35 compared to the control (0/7 mice with reduced tumor growth). However, after observing the mice for 49 days, 4/7 mice treated with V-scVLPs survived beyond 49 days (compared to none in the control) (Figure 4D), although these mice still had tumors with high luciferase activity. The observed difficulty in completely suppressing established tumor growth in mice treated with V-scVLPs alone may be due to the presence of dysfunctional T cells and an immunosuppressive TME during tumor progression 44,63. Upregulation of TIM-3 expression on CD8^+^T cells is considered as a hallmark of T cell dysfunction within an immunosuppressive TME 64-66. Accordingly, V-scVLPs-generated CD8^+^T cells exhibited upregulated TIM-3 expression (18.84% ± 2.61%) in tumors compared to the control (9.80% ± 1.15%) (Figure S23-24). These results suggest limitations and challenges associated with V-scVLPs treatment alone in an immunosuppressive TME. We then investigated whether the combination of V-scVLPs with the αTIM-3 antibody could enhance their antitumor efficacy. When comparing with the efficacy of αTIM-3 therapy alone or V-scVLPs treatment alone, we found that the combination therapy resulted in a dramatically enhanced anti-tumor effect (Figure S25).

Monitoring the orthotopic tumor luciferase activity showed that the combination therapy significantly reduced the signal at day 21 (5/7 mice) and day 42 (4/7 mice), as well as at the end of observation day 49 (3/7 mice) (Figure 4D). Furthermore, the combination therapy extended the survival time of 5/7 mice beyond 49 days, whereas none of the mice treated with αTIM-3 alone (0/7) survived beyond 49 days. The combination therapy also effectively suppressed liver cancer lung metastases in 3/7 mice, in contrast to the αTIM-3 (0/7 mice) or V-scVLPs (1/7 mice) treatment alone (Figure 4E and S26). Furthermore, to confirm the synergistic effect of V-scVLPs and αTIM-3 therapy in mice, we calculated the synergistic effect using the Bliss independent model 58. Our results showed that the* Y_ab,O_* value of the combined treatment was 0.857, which was higher than the value of V-scVLPs or α-TIM-3 treatment alone (*Y_ab,P_*, 0.796), indicating a synergistic effect of V-scVLPs and α-TIM-3 therapy in suppressing tumor growth. These results demonstrated that combination with anti-TIM-3 therapy could reactivate and enhance the tumor-killing capacity of CD8^+^ effector T cells to significantly inhibit the orthotopic HCC tumor growth and liver cancer lung metastases.

To gain further insight into the effect of combination treatments on the intrahepatic immune microenvironment, we assessed the population of regulatory T cells (Tregs) within the tumors by FCM. As shown in Figure 4F-G and S27, treatment with V-scVLPs or the combination therapy with αTIM-3 antibody efficiently reduced the number of Foxp3^+^CD25^+^T cells (Tregs^V-scVLPs^, 4.35%± 2.04%; Tregs^combination treatments^, 4.25%±0.99%) compared to the control. In addition, tissue-resident memory CD8^+^T cells expressing CD69^+^ in the tumor were higher in the combination treatment group (10.37% ± 1.41%) than in V-scVLPs treatment group (8.46% ± 0.80%) or αTIM-3 treatment alone (6.52% ± 1.27%), which correlated with improved survival during αTIM-3 therapy as previously reported 67 (Figure 4H-I, S28). Furthermore, we observed a significantly higher number of tumor-reactive IFNγ^+^CD8^+^T-cells and increased expression of the 41BB co-stimulatory receptor on CD8^+^T cells in the combination treatment group compared to the other treatments (Figures 4J-M, S29-30). These results demonstrated that the combination therapy was able to elicit a potent activation of CD8^+^ effector T cells.

Furthermore, tumor-derived cytokine levels measured by ELISA kits showed that the combination therapy with αTIM-3 indeed increased the IFNγ, TNF-α, IL-2, IL-6 and IL12 secretion to induce potent anti-tumor immunity to suppress tumor progression comparing with αTIM-3 therapy alone or V-scVLPs treatment alone (Figure 4N). Immunofluorescence assays showed that a higher number of infiltrating CD8^+^T cells (red) were observed in tumors after the combined treatments compared to the αTIM-3 or V-scVLPs treatments alone (Figure 4O). Moreover, a larger number of apoptotic cells (green) with the highest TUNEL fluorescence signal and lowest Ki67 signal (brown) were observed in the orthotopic tumors after the combined treatments. In contrast, only a few apoptotic cells with lower TUNEL signal were detected in both V-scVLPs treatment and anti-TIM-3 therapy alone. These results demonstrate that the combination therapy with αTIM-3 effectively alleviated the exhausted CD8^+^T-cell functions induced by V-scVLPs and promoted effector CD8^+^T-cell infiltration into orthotopic tumors to effectively suppress tumor growth and lung metastasis of liver cancer.

**Figure 5:**
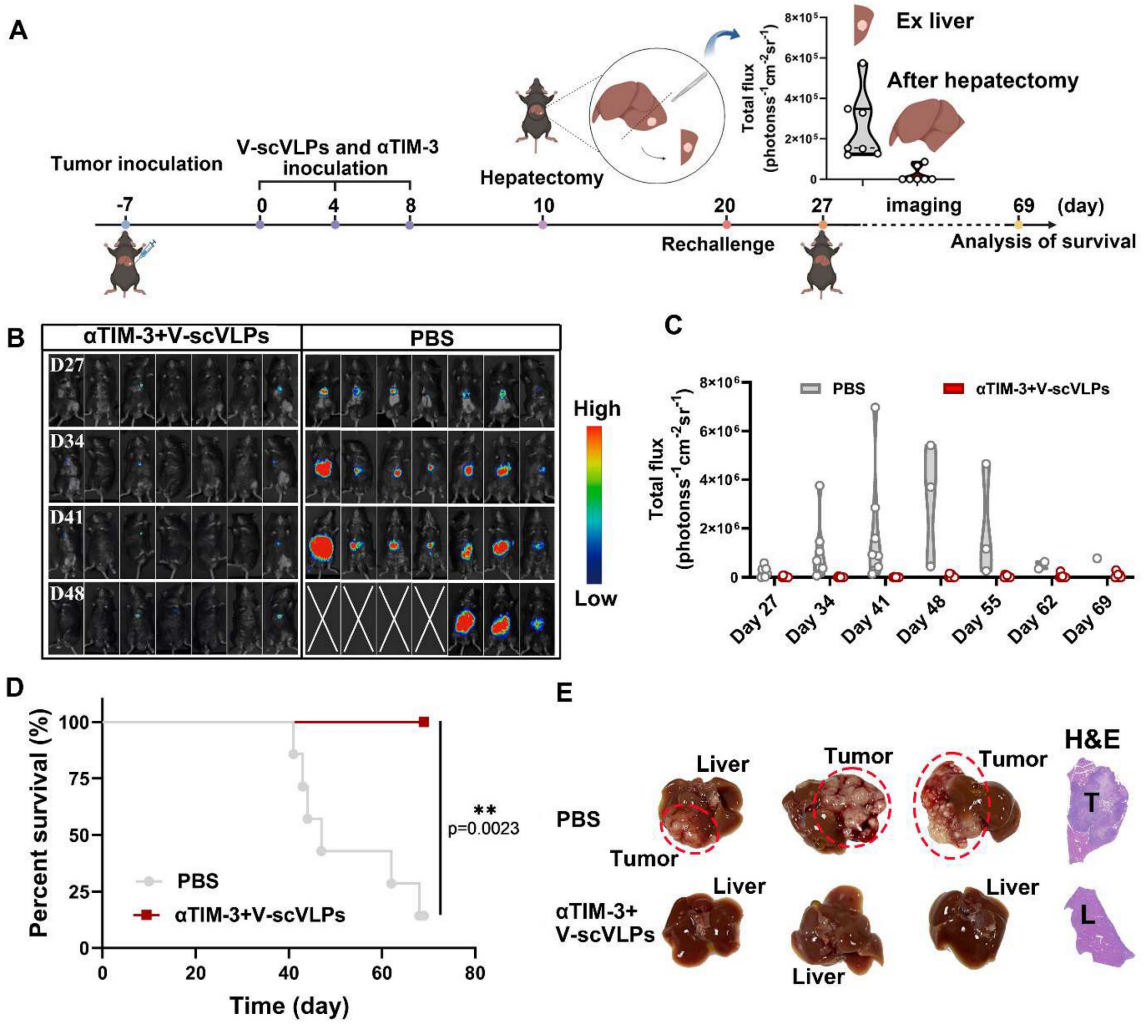
Suppression of tumor recurrence after hepatectomy. (A) Schematic illustration of the tumor re-challenge process under combined treatment in established orthotopic Hepa1-6-luc tumor model after hepatectomy. Total flux intensity of *ex*-tumors isolated from hepatectomy and mice after hepatectomy, (n = 7). (B) Bioluminescence imaging and (C) total flux intensity of orthotopic Hepa1-6-luc bearing mice and total flux at 0, 14, 21 and 28 days after receiving different treatments as indicated. (n = 7). (D) The survival curve of orthotopic Hepa1-6-luc bearing mice after indicated treatment (n = 7). (E) Photomicrograph and H&E staining of orthotopic liver tumor from one of the mice that received PBS, V-scVLPs or Neo/ODN treatment, respectively. Statistical analysis was performed with ANOVA analysis, *p<0.05, **p<0.01, ***p<0.001, ****p<0.0001. Data are expressed as mean ± SD.

To realistically simulate the clinic and to investigate the inhibition of tumor recurrence of this combined therapeutic strategy, the established orthotopic HCC mice that received the combined treatment were underwent hepatectomy (right lobe of the liver) after 10 days of treatment. Successful hepatectomy was confirmed by photographing the liver tissue, H&E staining and luciferase activity of the mice (Figure 5A and S31). The Hepa1-6-luc cells were then intra-hepatically inoculated into the left lobe of the liver to simulate tumor recurrence. Interestingly, the results showed that the combined treatment was able to effectively and even completely eliminate the inoculated Hepa1-6-luc cells in 5/7 of the mice on the day 27. These mice still exhibited the lowest luciferase activity on the day 48 compared to the PBS-treated mice with the highest luciferase activity (Figure 5B-C). In addition, the combination treatment was more efficient in prolonging the survival of mice (7/7) beyond 69 days, while only one out of seven mice survived beyond 69 days without any treatment (Figure 5D and S32). These results suggest that the combined treatment is a promising approach for the treatment of liver cancer. Photomicrographs of *ex-*liver and H&E sections further confirmed above results (Figure 5E, S33). Besides, the negligible weight loss of the mice during the treatments, along with the serological and physiological biochemical index (including ALT, AST, and ALP, etc.), and histological examination using H&E staining of the major organs, indicated the biosafety of V-scVLPs and the combined treatment strategy (Figure S34-36). These results demonstrated that the combined treatment could generate a long-lasting memory to efficient inhibit tumor recurrence and prolong the survival of the mice, providing an efficient therapeutic strategy to improve HCC immunotherapy.

## Conclusion

In conclusion, we successfully constructed a non-viral scVLPs carrier for efficient delivery of NeoAgs vaccine *via* caveolin-mediated endocytosis to enhance innate and adaptive immunotherapy. The V-scVLPs, composed of scVLPs, ODN-1826 and NeoAgs, achieved the effective retention and lymph node homing *in vivo*. The superior intracellular delivery and lymph node homing of V-scVLPs significantly enhanced IFNγ^+^CD8^+^T-cell infiltration and T_CM_ memory phenotype, which prevented the orthotopic tumor growth and prolonged animal survival in a prophylactic setting. Importantly, the combination of anti-TIM-3 therapy and V-scVLPs rescued the exhausted CD8^+^T cell antitumor functions in a therapeutic setting, while reduced the Tregs population and improved the established intrahepatic tumor immune microenvironment to effectively suppress tumor growth, liver cancer lung metastasis, recurrence after hepatectomy, then prolonged animal survival. Taken together, this study proposes a promising neoantigen nanovaccine for future clinical translation to improve HCC immunotherapy.

## Supplementary Material

Supplementary table and figures.
