# Supplementary material for: Caveolin-mediated cytosolic delivery of spike nanoparticle enhances antitumor immunity of neoantigen vaccine for hepatocellular carcinoma
Source: Theranostics. 2023 Jul 16;13(12):4166–81. doi: 10.7150/thno.85843 (PMC10405843; doi:10.7150/thno.85843)
Supplement: Supplementary file 1 — Supplementary table and figures. [file thnov13p4166s1.pdf]

Supplementary information

Caveolin-mediated cytosolic delivery of spike nanoparticle enhances antitumor immunity of neoantigen vaccine for hepatocellular carcinoma

Zhiwen Lin[1, 2#], Chenwei Jiang[1, 2#], Peiyuan Wang[3#], Qingjing Chen[1], Bing Wang[1], Xinyue Fu[1], Yuzhi Liang[1,5], Da Zhang[1, 4*], Yongyi Zeng[1,2*], Xiaolong Liu[1,3,4*]

[1.] The United Innovation of Mengchao Hepatobiliary Technology Key Laboratory of Fujian Province, Mengchao Hepatobiliary Hospital of Fujian Medical University, Fuzhou 350025, P. R. China
[2.] Liver Disease Center, The First Affiliated Hospital of Fujian Medical University, Fuzhou 350005, People's Republic of China
[3.] CAS Key Laboratory of Design and Assembly of Functional Nanostructures, Fujian Institute of Research on the Structure of Matter, Chinese Academy of Sciences, Fuzhou 350002, P. R. China
[4.] Mengchao Med-X Center, Fuzhou University, Fuzhou 350116, P. R. China
[5.] Fujian Agriculture and Forestry University, Fuzhou 350002, P. R. China

#These authors contributed equally to this paper

*Corresponding authors:

E-mail addresses: Prof. Da Zhang (zdluoman1987@163.com), Prof. Yongyi Zeng (lamp197311@126.com) and Prof. Xiaolong Liu (xiaoloong.liu@gmail.com).

Table S1. Encapsulation efficiency and loading capacity of NeoAgs and ODN-1826 in V-scVLPs and SPS, respectively.

scVLPs	NeoAgs	ODN-1826
Entrapment efficiency	91.8%	76.6%
Loading efficiency	31.46%	4.57%

SPS	NeoAgs	ODN-1826
Entrapment efficiency	63.1%	38.8%
Loading efficiency	23.98%	2.37%

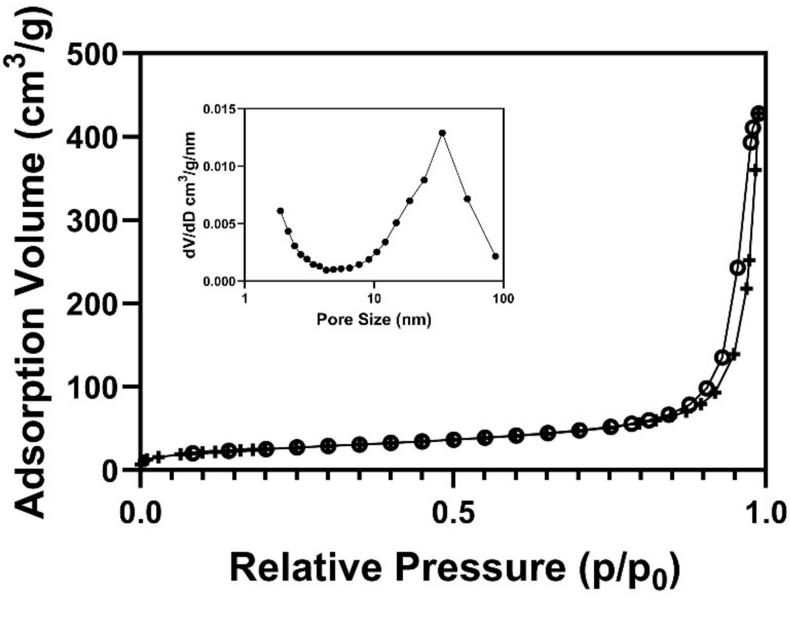

Figure. S1. N2 sorption isotherm for scVLPs and DFT pore size distribution for scVLPs.

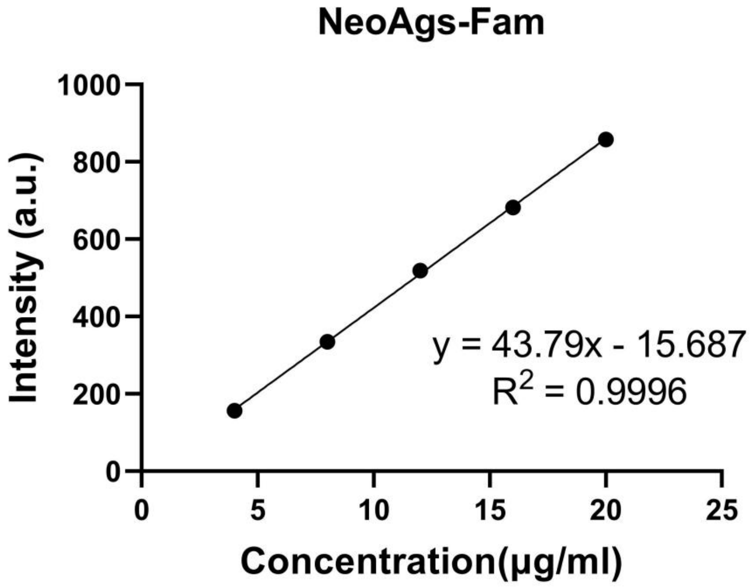

Figure. S2. The concentration of NeoAgs from scVLPs ($^{FAM-}$NeoAgs) was quantified according to the linear fit (Y = 43.79x -15.687, R^2 = 0.9996) of fluorescence intensity at 520 nm, the correlation curve was prepared from 4.0~20.0 μg/mL.

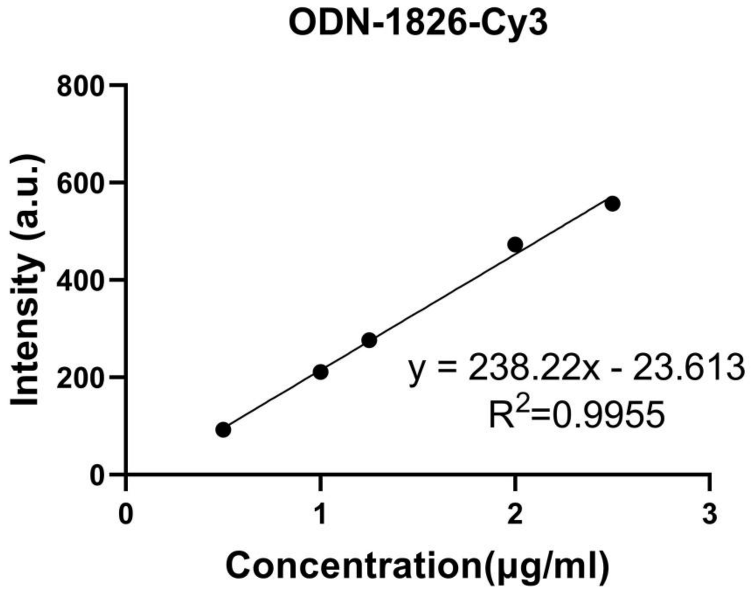

Figure. S3. The concentration of ODN-1826 from scVLPs was quantified according to the linear fit (Y = 238.22x -23.613, R^2 = 0.9955) of fluorescence intensity at 570 nm, the correlation curve was prepared from 0.5~2.5 μg/mL.

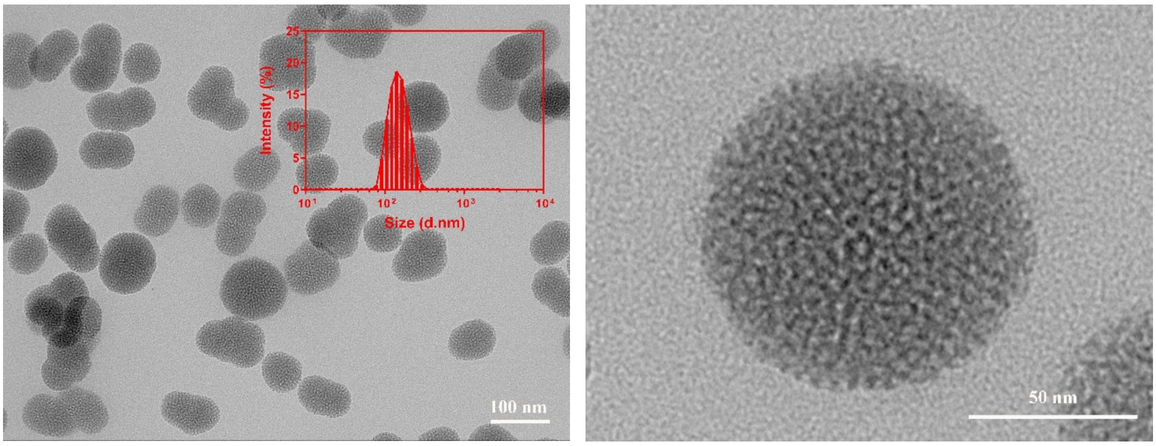

Figure. S4. TEM image of SPS.

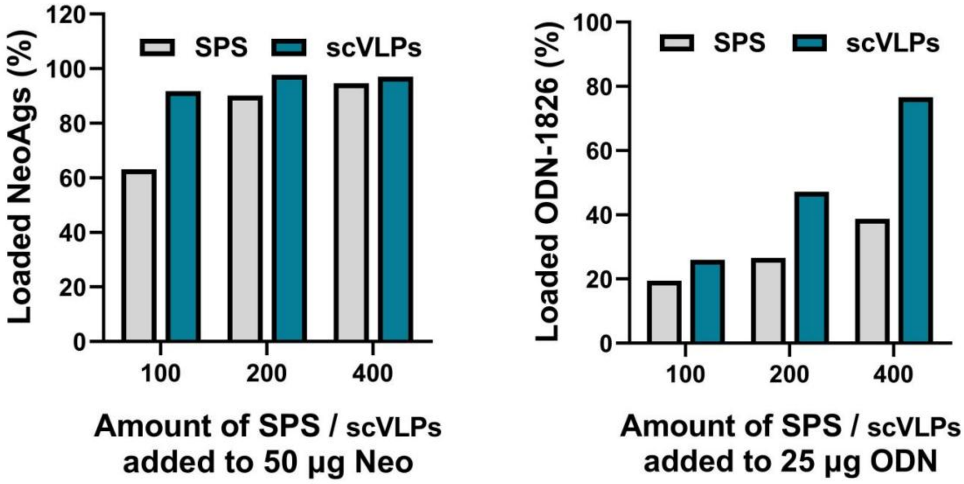

Figure. S5. The loading percentage of NeoAgs and ODN-1826 in SPS or scVLPs, respectively.

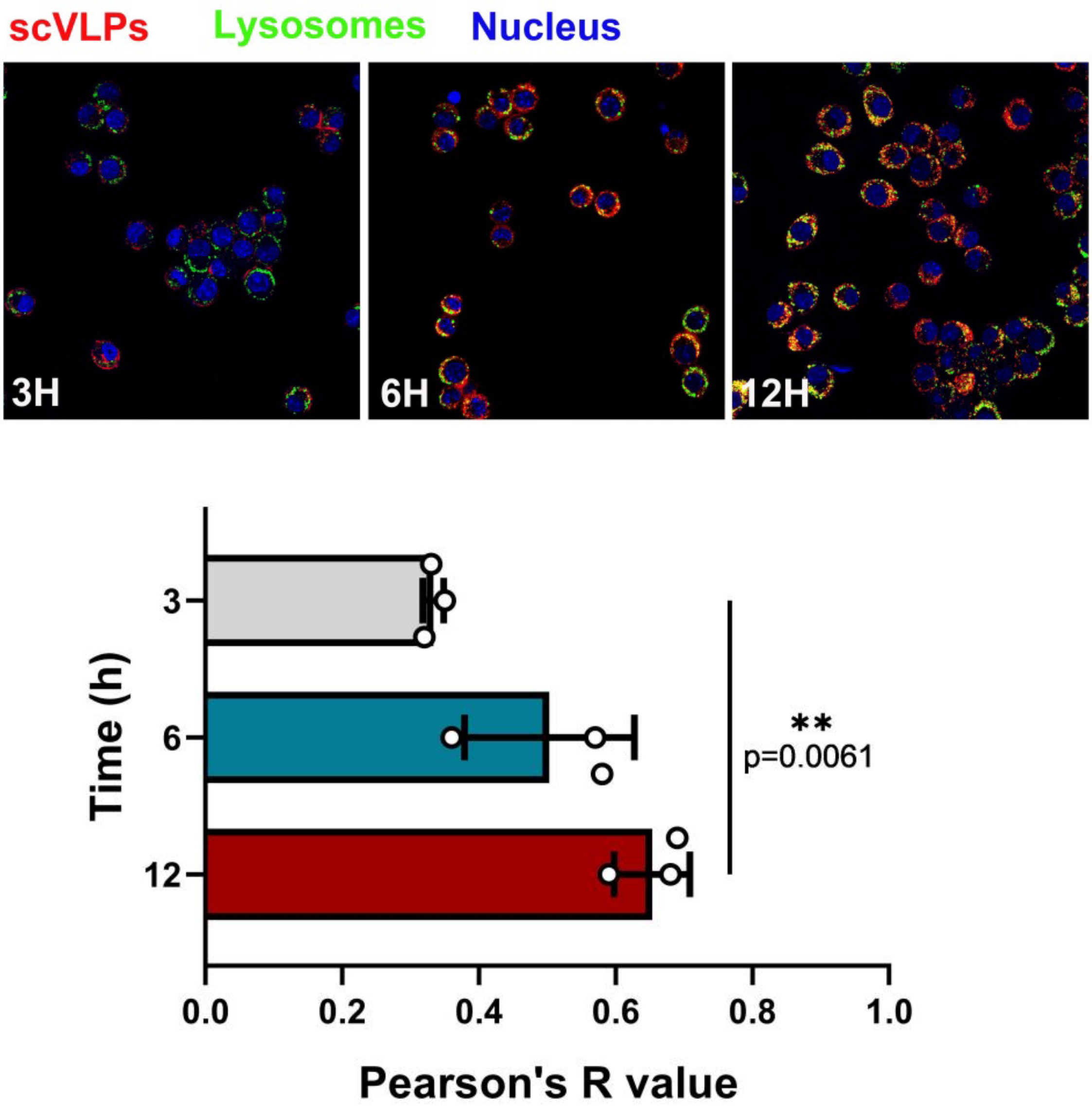

Figure. S6. PCCs of Cy5 labeled scVLPs with lysosomes at different incubation times, (n=3). The statistical analysis was performed with ANOVA analysis, *$p<0.05$, **$p<0.01$, ***$p<0.001$, ****$p<0.0001$*, (n = 3). Data are presented as mean ± SD.

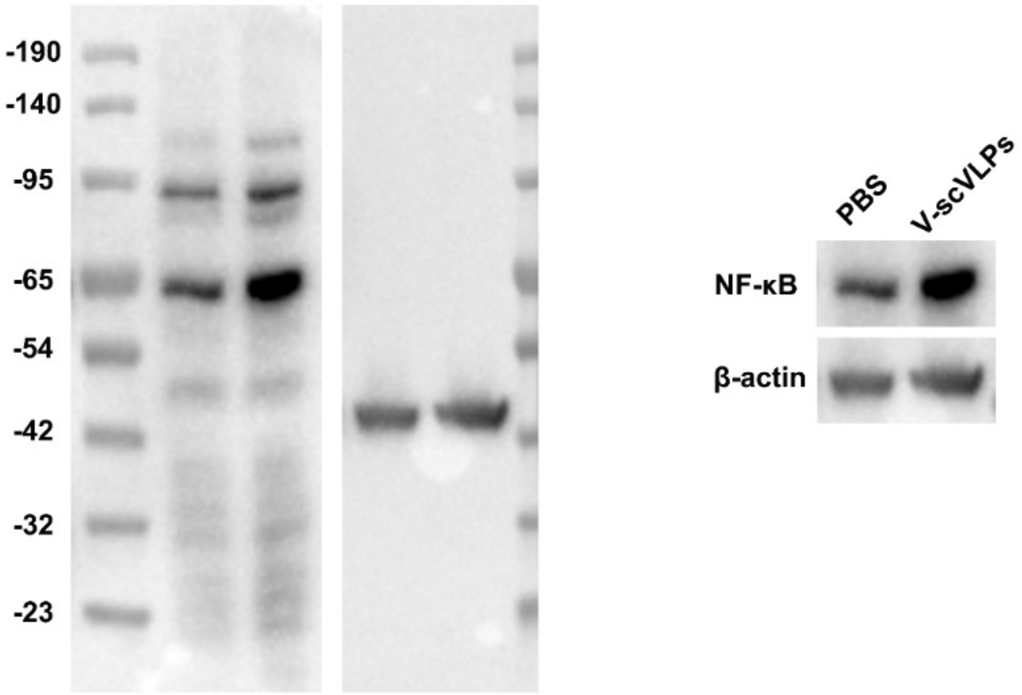

Figure. S7. Western blot analysis of NF-kB in BMDCs after co-incubation with V-scVLPs for 48 h, and PBS treated cell were used as control.

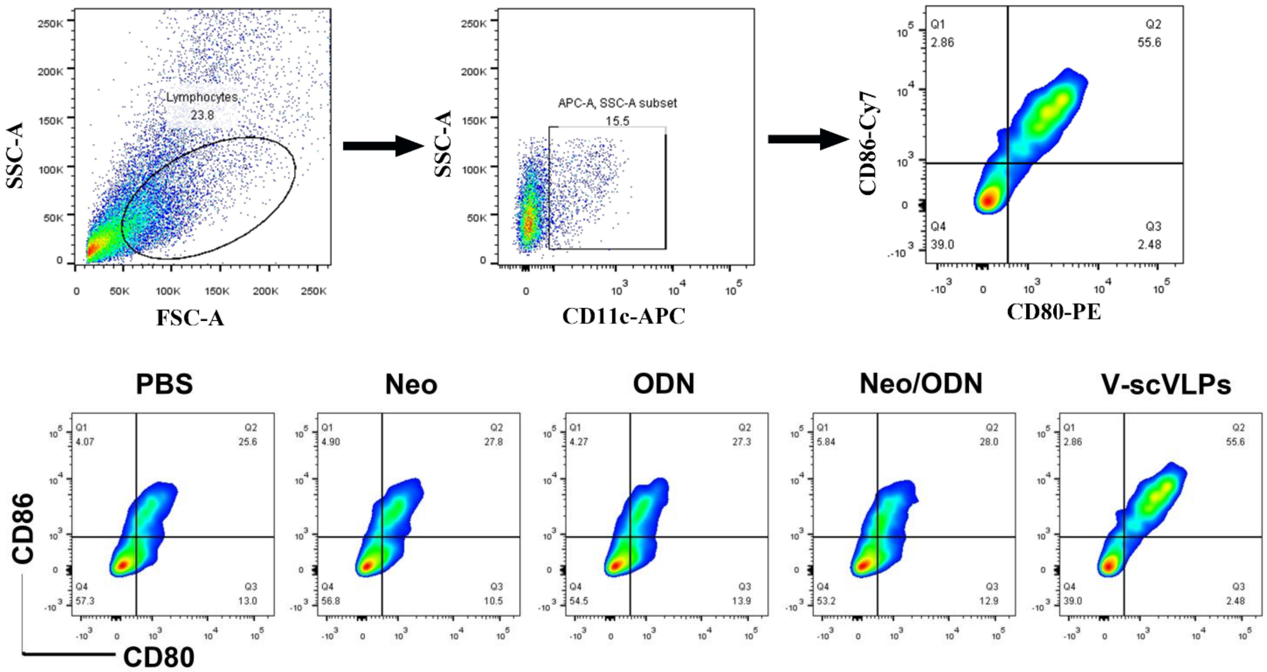

Figure. S8. Identify the maturation of BMDCs after receiving different treatment through FACS with staining anti-CD11c-APC, anti-CD80-PE, and anti-CD86-PE-Cy7 antibodies.

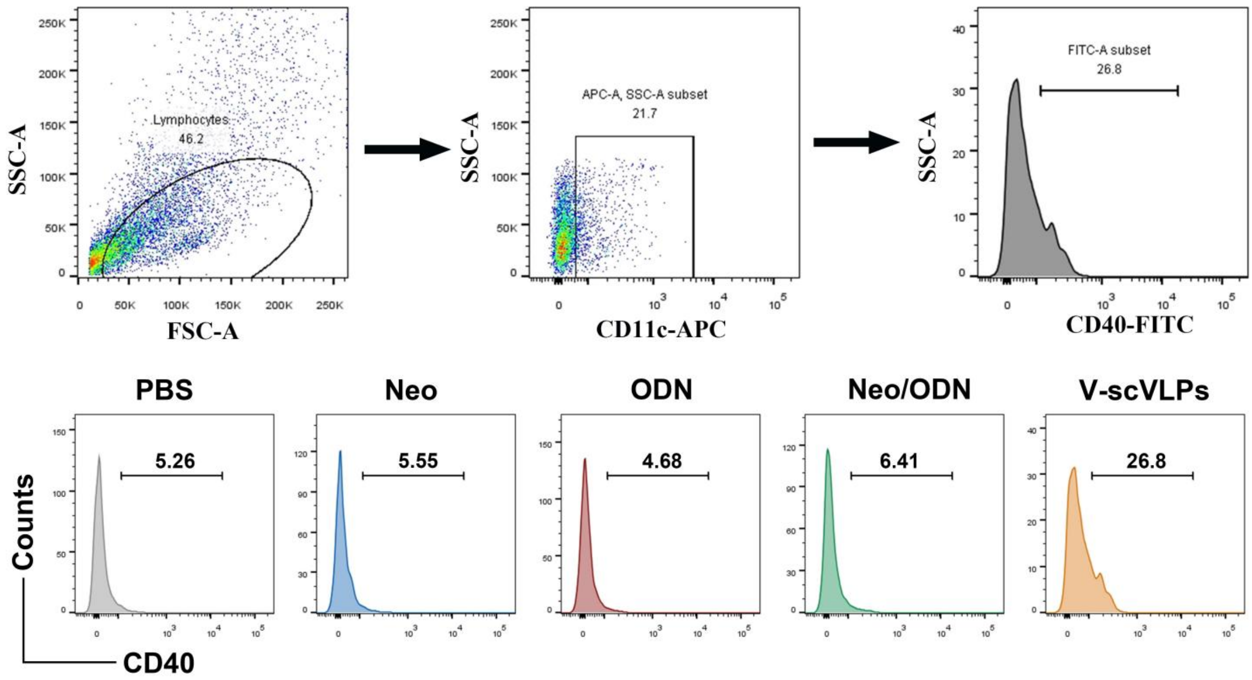

Figure. S9. Identify the maturation of BMDCs after receiving different treatment through FACS with staining anti-CD11c-APC, anti-CD40-FITC antibodies.

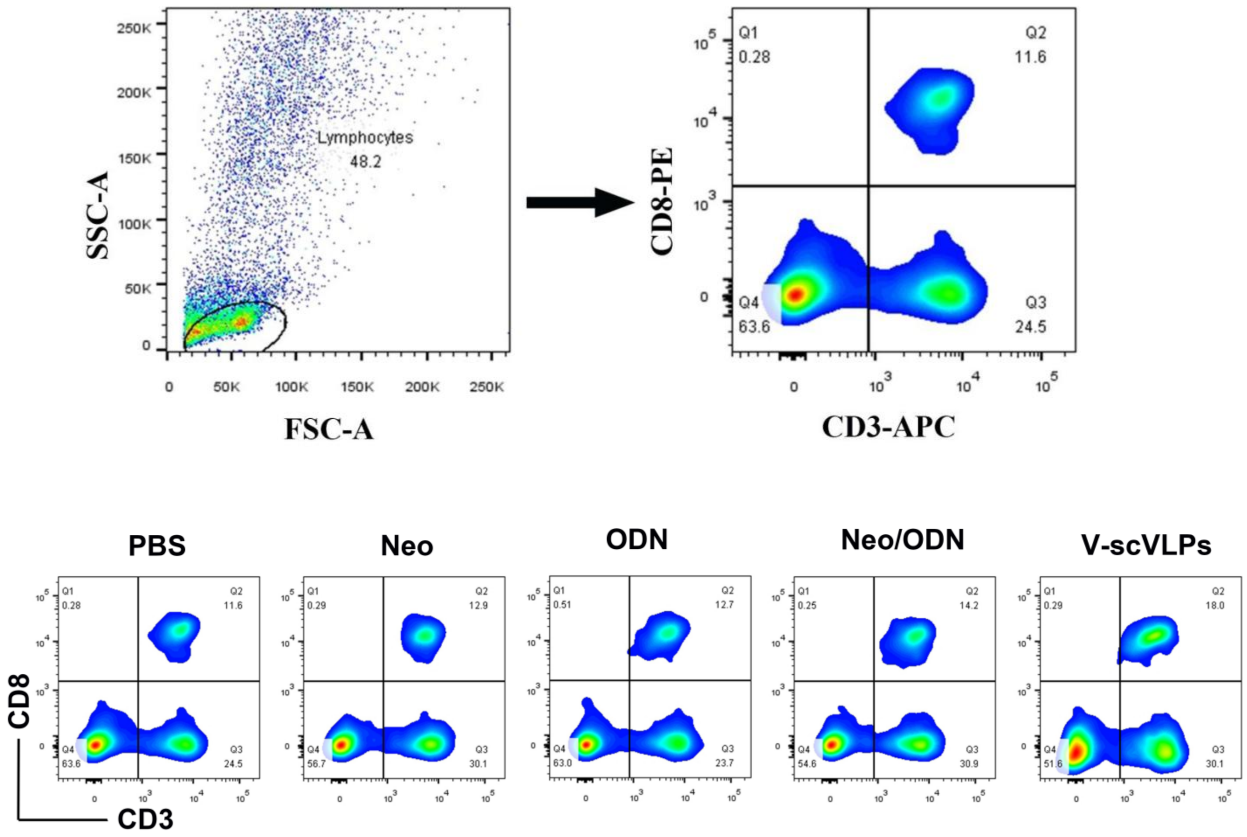

Figure. S10. Identify the population of T cells after receiving different treatment through FACS with staining anti-CD3-APC, anti-CD8-PE antibodies.

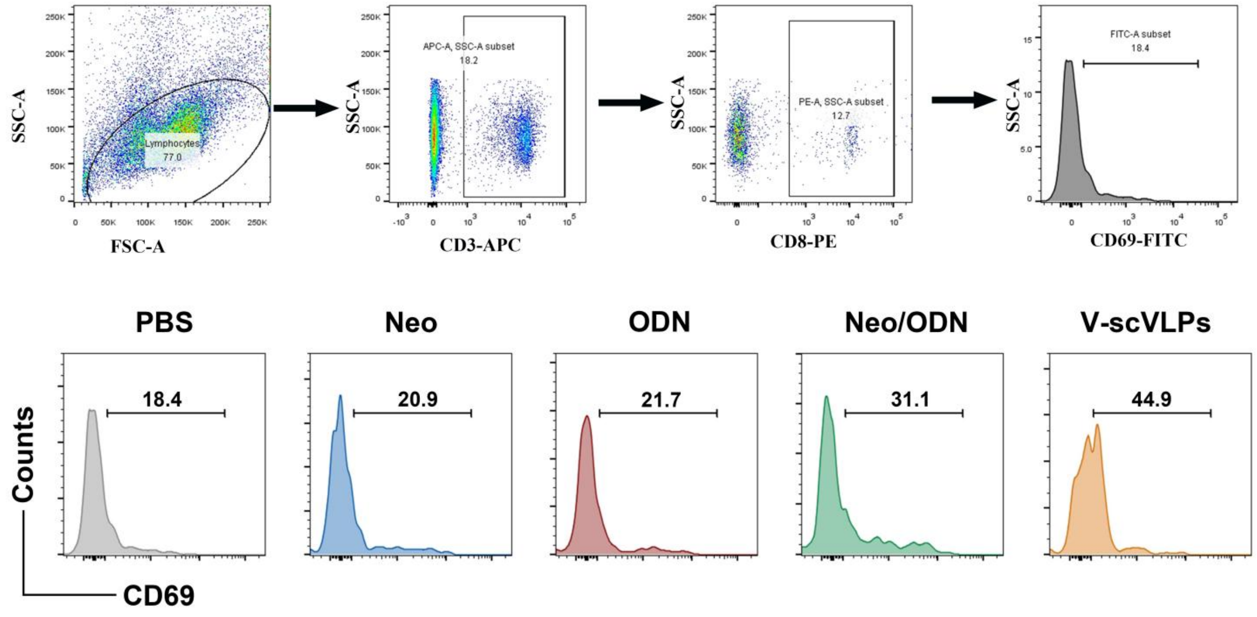

Figure. S11. Identify the activation of T cells after receiving different treatment through FACS with staining anti-CD3-APC, anti-CD8-PE and anti-CD69-FITC antibodies.

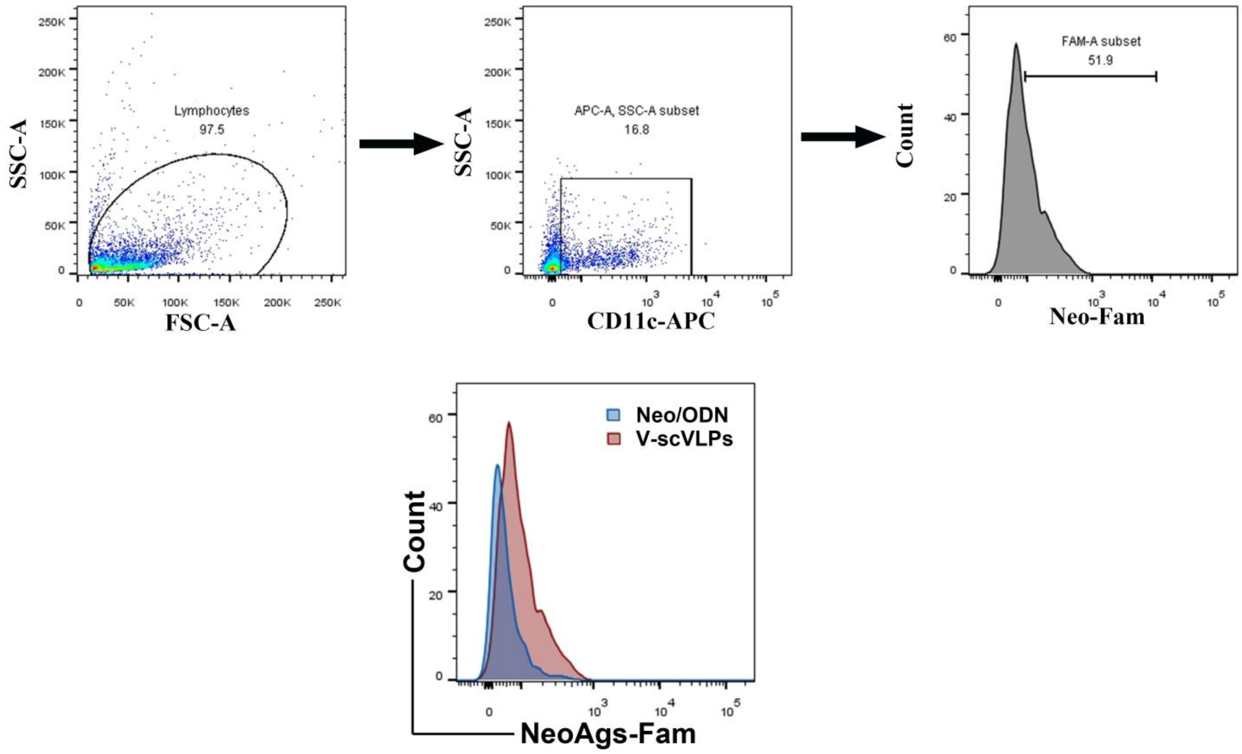

Figure. S12. Representative fluorescence intensity of ^{FAM}NeoAgs positive cells gated on DCs in lymph node after 12h of Neo/ODN and scVLPs injection.

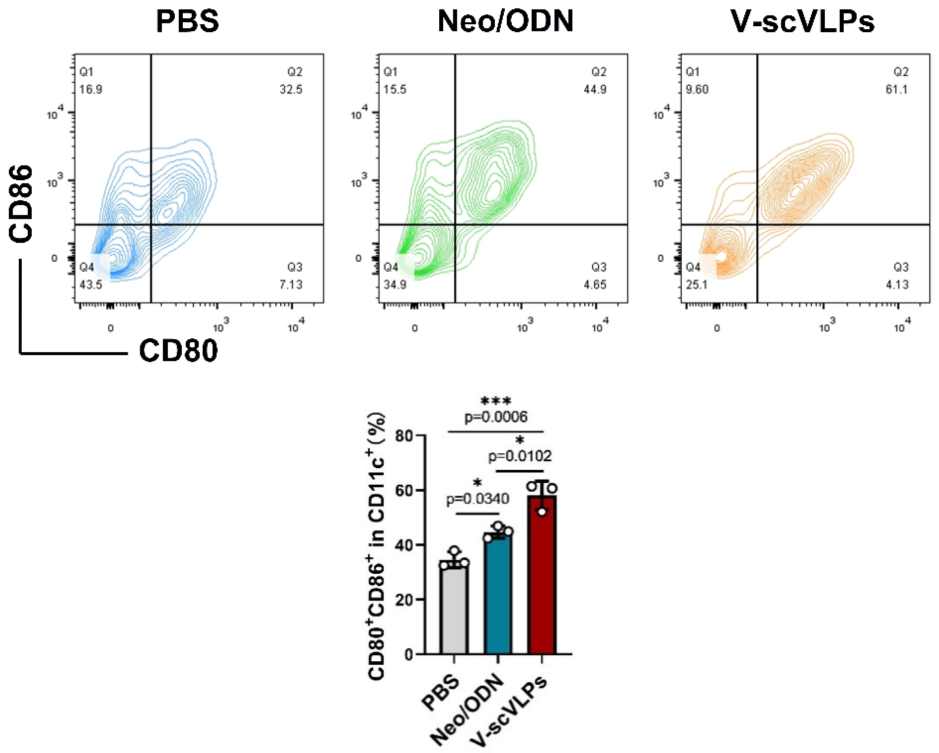

Figure. S13. Identify the maturation of DCs in LNs after receiving different treatment through FACS with staining anti-CD11c-APC, and anti-80-PE and anti-86-PECy7 antibodies.

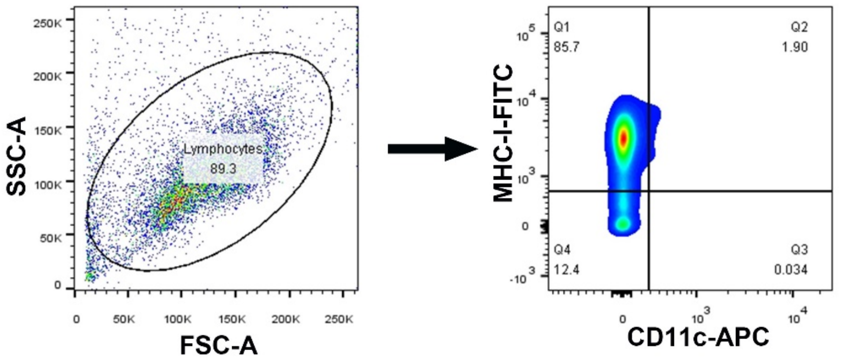

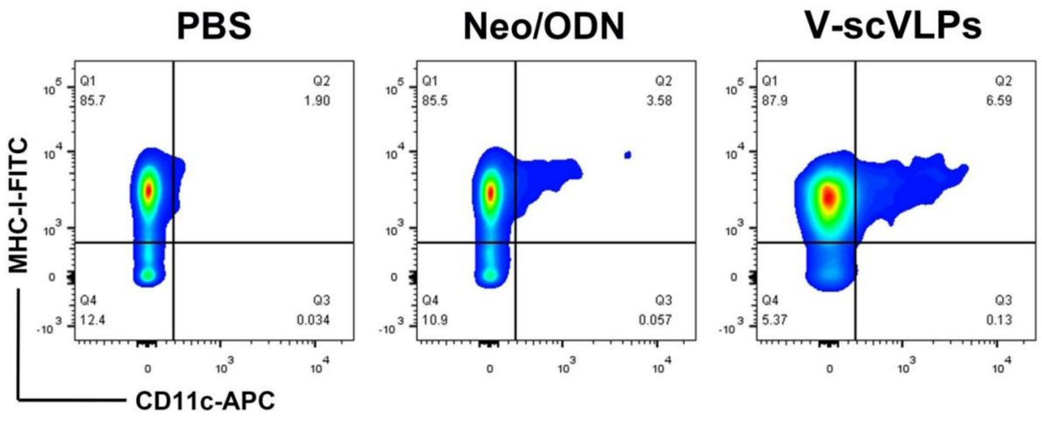

Figure. S14. Identify the maturation of DCs in LNs after receiving different treatment through FACS with staining anti-CD11c-APC, and anti-MHC-I-PE antibodies.

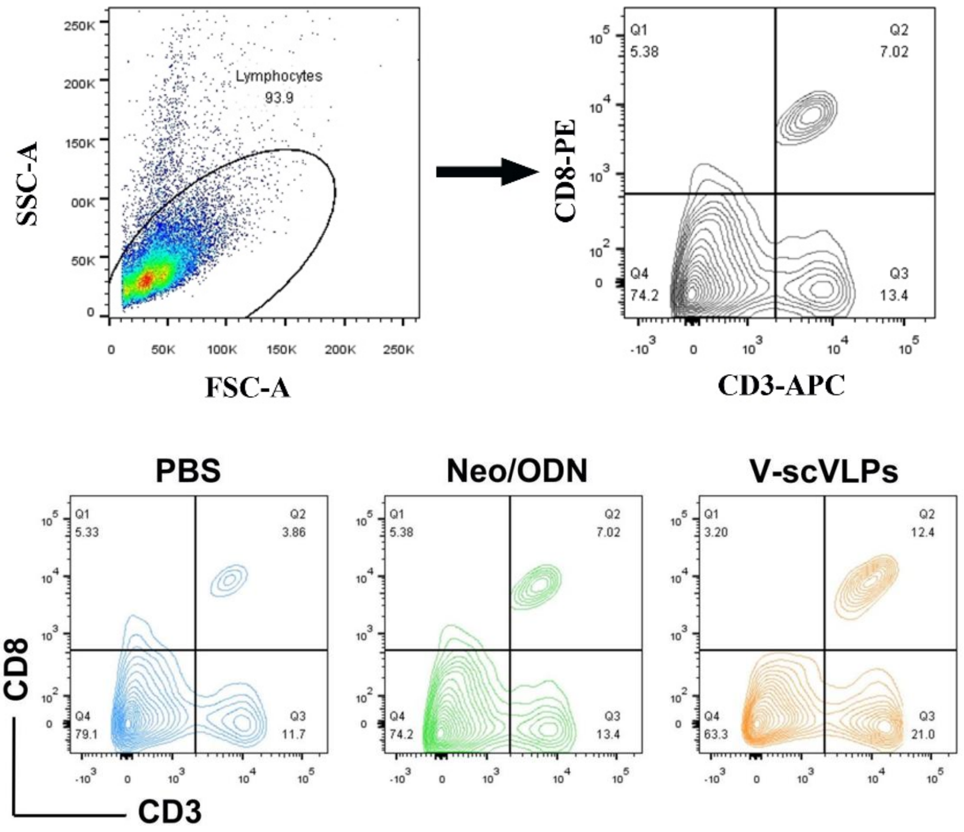

Figure. S15. Identify the $CD8^{+}$T cells in spleen after receiving different treatment through FACS with staining anti-CD3-APC and anti-CD8-PE antibodies.

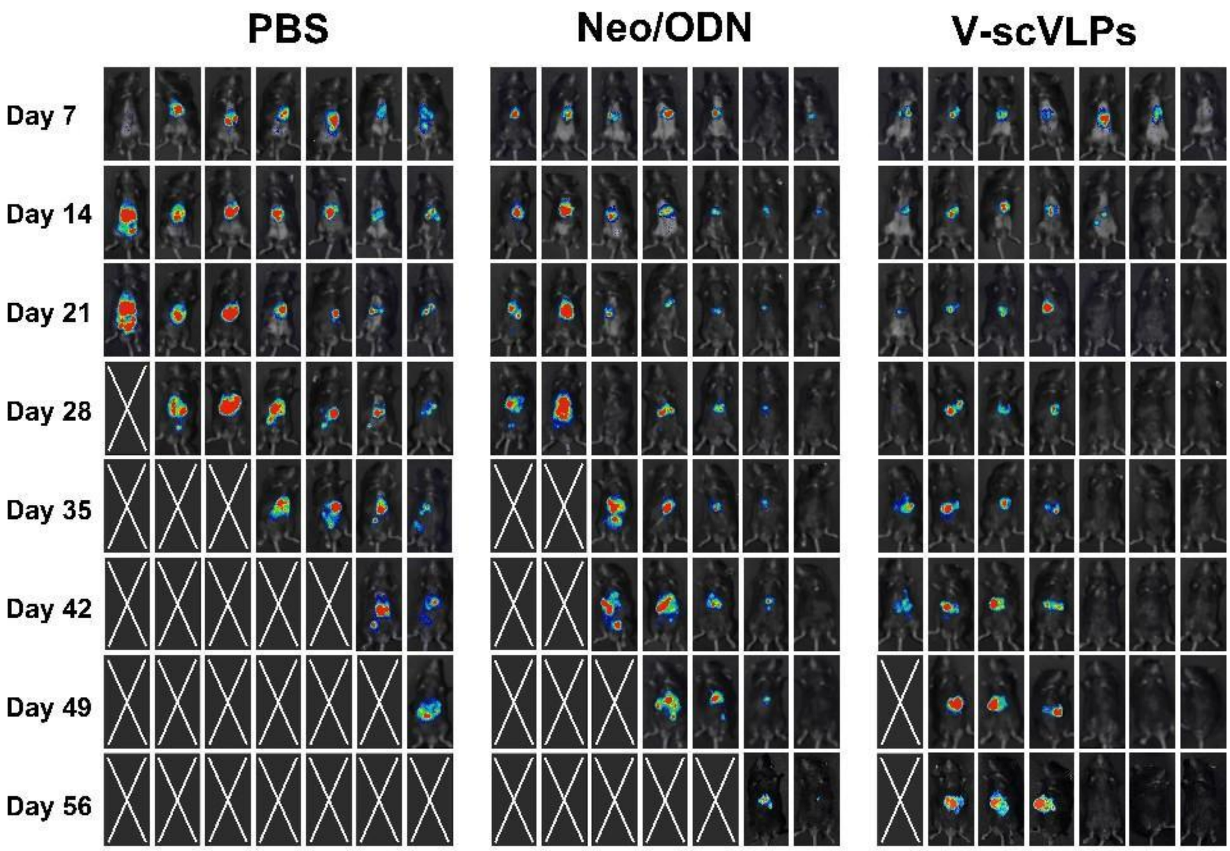

Figure. S16. Bioluminescence imaging of mice at the 7, 14, 21, 28, 35, 42, 49 and 56 days before and after receiving different treatments as indicated, n = 7.

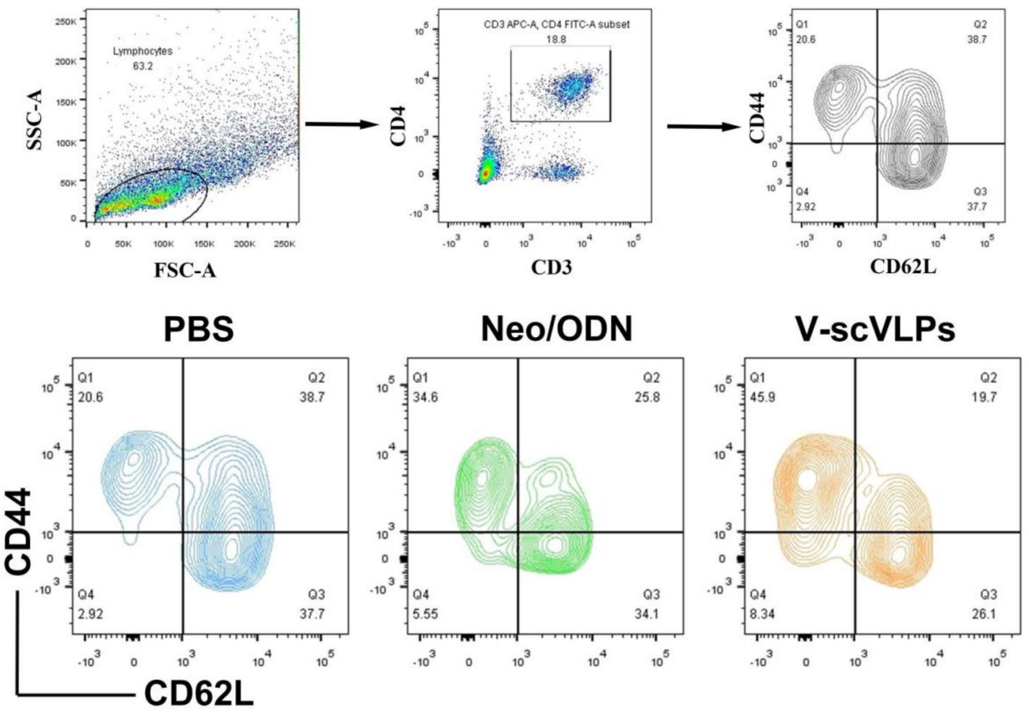

Figure. S17. Identify the CD3⁺CD4⁺CD44⁺CD62L⁺T cells in the spleen after receiving different treatments through FACS with staining anti-CD3-APC, anti-CD4-FITC antibodies, anti-CD44-PE-Cy7 and anti-CD62-PerCP-Cy5.5 antibodies.

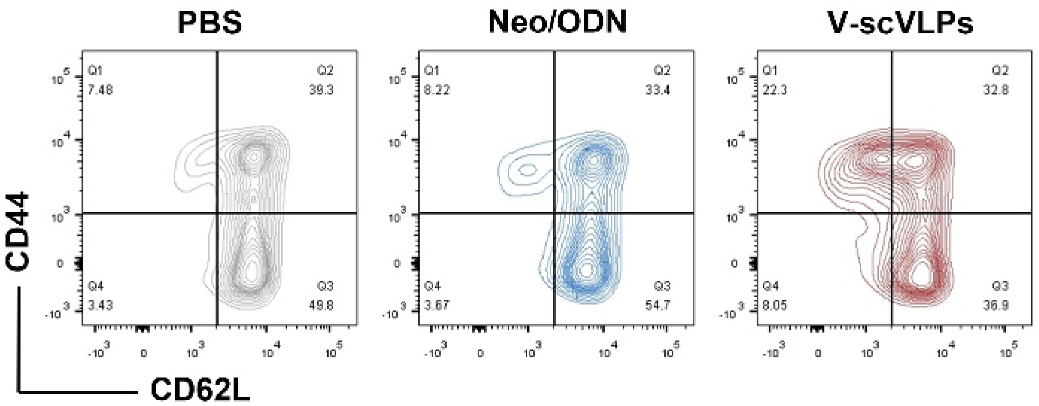

Figure. S18. Identify the CD3⁺CD8⁺CD44⁺CD62L⁺T cells in the spleen after receiving different treatments through FACS with staining anti-CD3-APC, anti-CD8-PE antibodies, anti-CD44-PE-Cy7 and anti-CD62-PerCP-Cy5.5 antibodies.

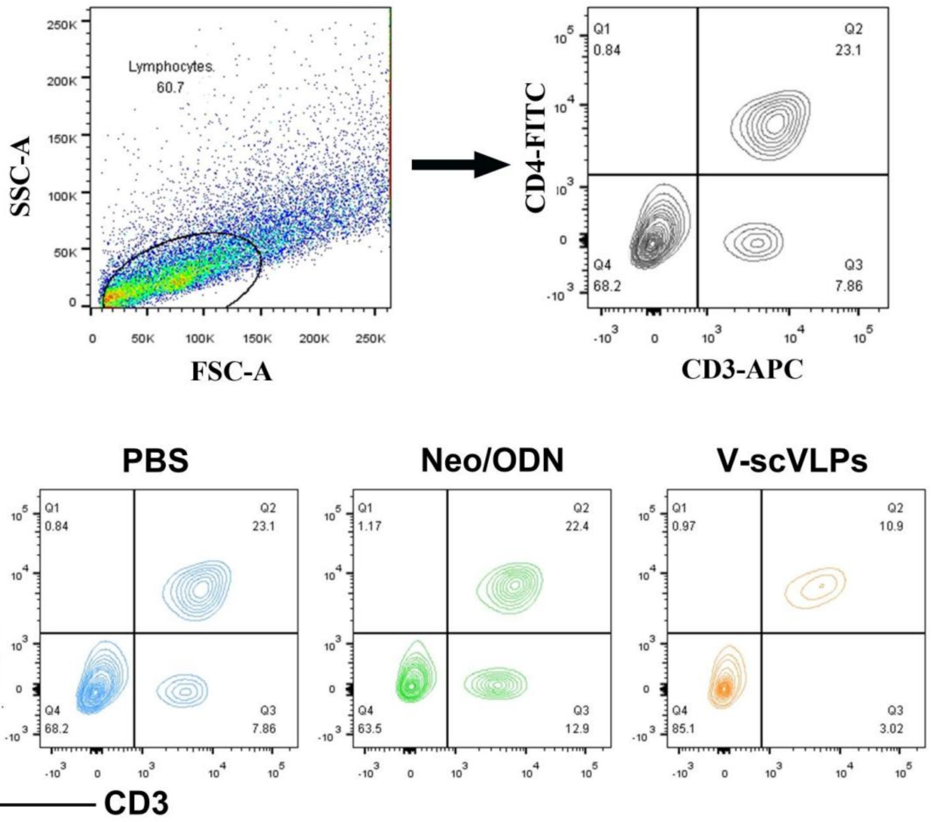

Figure. S19. Identify the $CD3^{+}CD4^{+}$T cells in the spleen after receiving different treatments through FACS with staining anti-CD3-APC and anti-CD4-FITC antibodies.

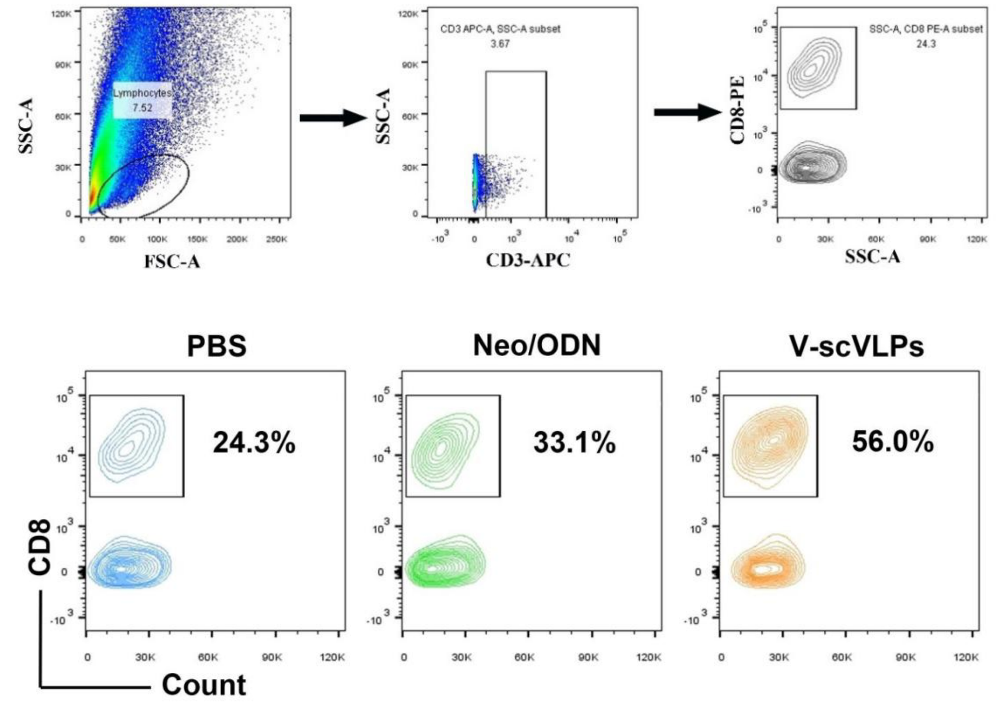

Figure. S20. Identify the CD8$^+$T cells in tumor after receiving different treatment through FACS with staining anti-CD3-APC and anti-CD8-PE antibodies.

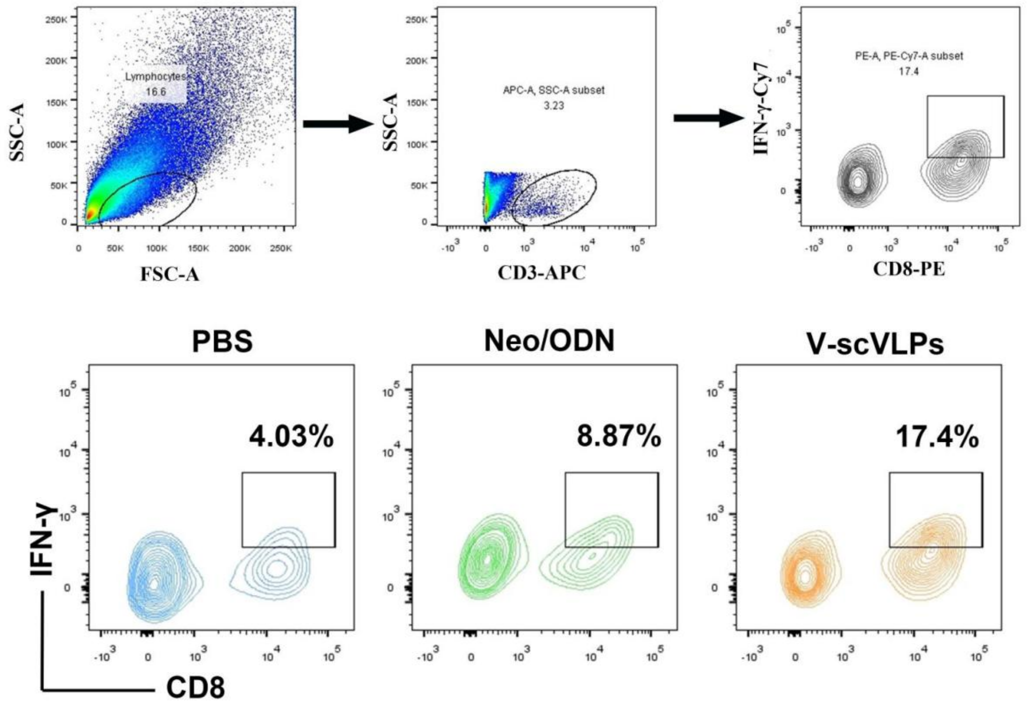

Figure. S21. Identify different types of IFN-γ$^+$CD8$^+$CD3$^+$T cells in tumors after receiving different treatments through FACS with staining anti-CD3-APC, anti-CD8-PE, IFN-γ- PE-Cy7 antibodies.

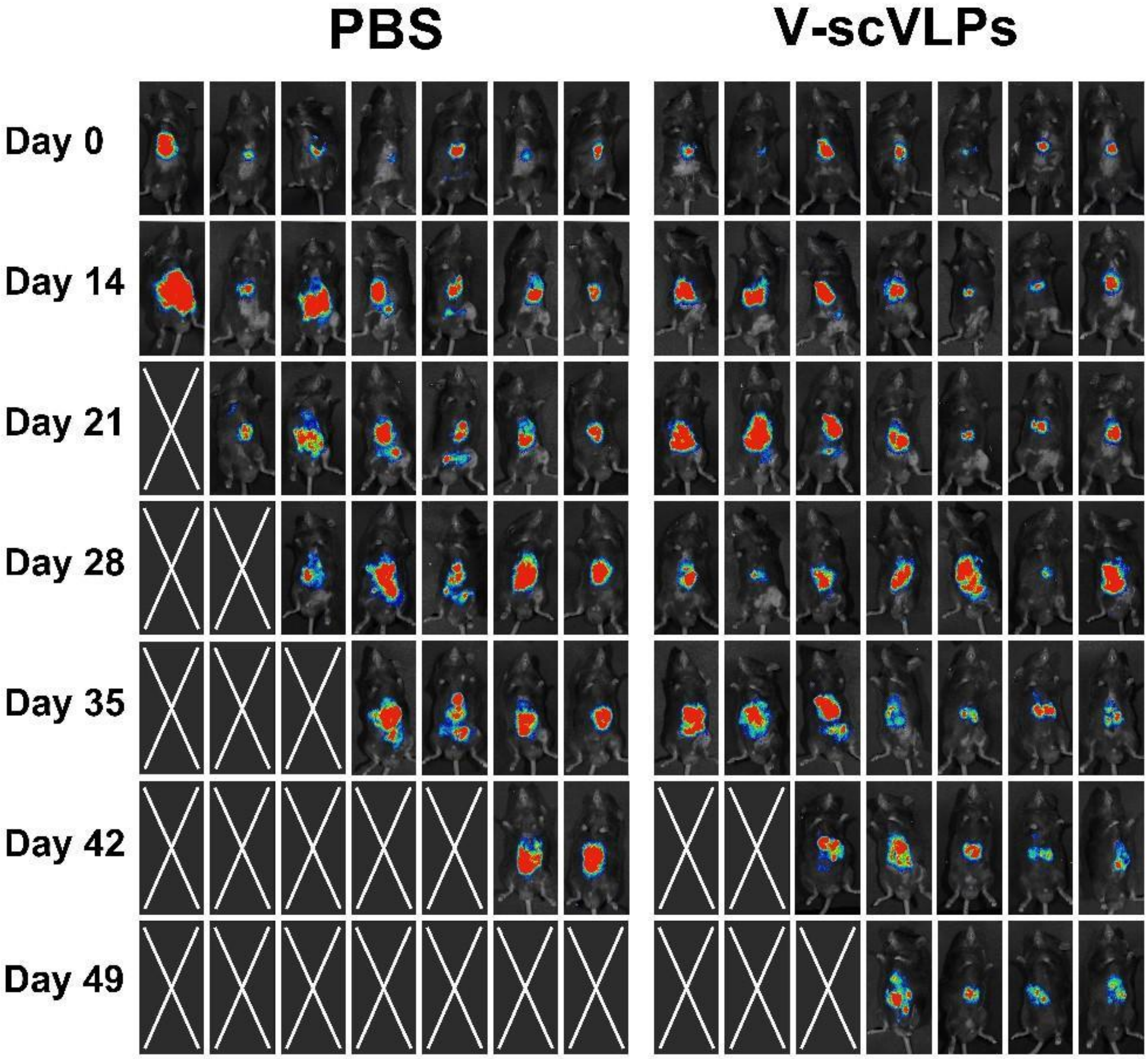

Figure. S22. Bioluminescence imaging of mice at the day 0, 14, 21, 28, 35,42 and 49 after receiving different treatments as indicated, n = 7.

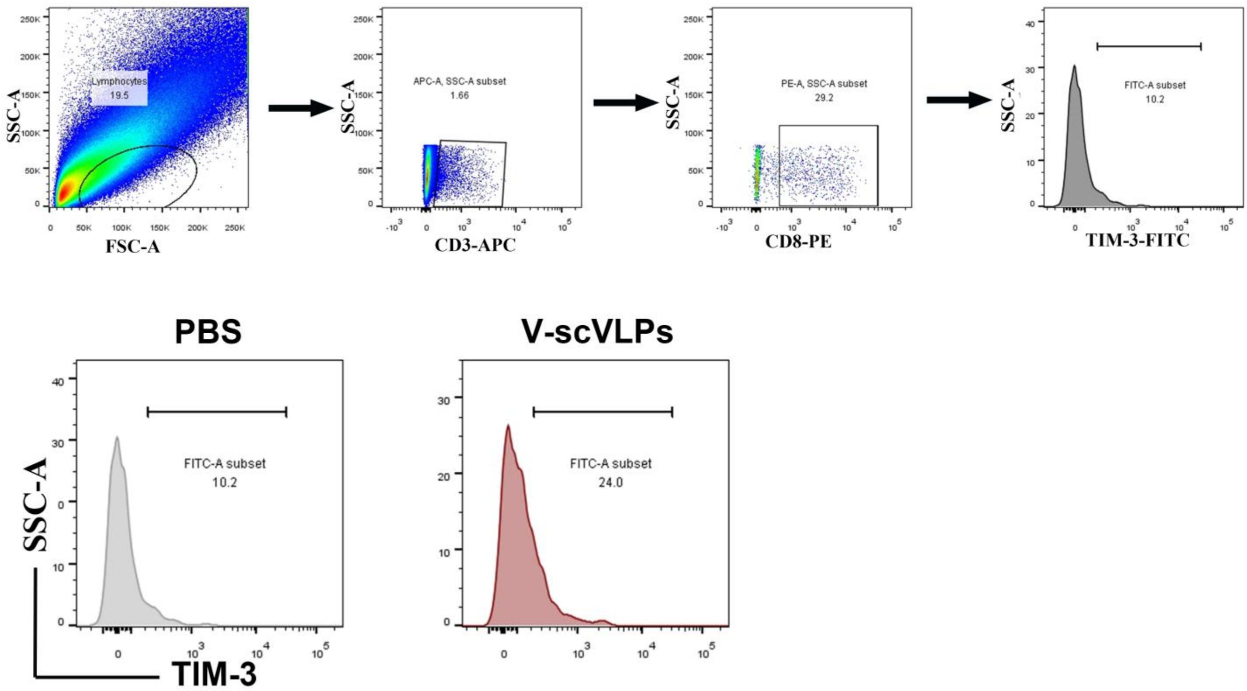

Figure. S23. Identify the different types of TIM-3$^+$CD8$^+$CD3$^+$T cells in tumors after receiving different treatments by FCM with staining anti-CD3-APC, anti-CD8-PE, TIM-3-FITC antibodies.

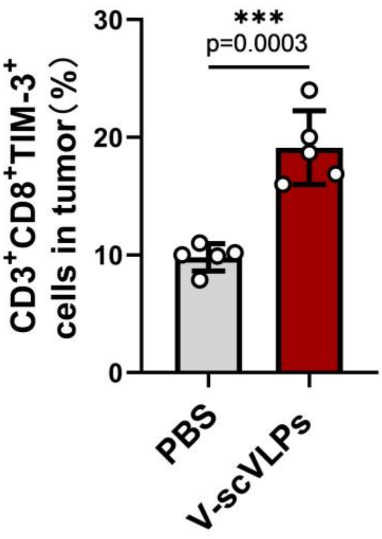

Figure. S24. The percentage of CD3$^+$CD8$^+$TIM-3$^+$T cells in orthotopic tumors after receiving different treatment as indicated, n = 5. Statistical analysis was performed with T test, **p<0.05, **p<0.01, ***p<0.001, ****p<0.0001*. Data are expressed as mean ± SD.

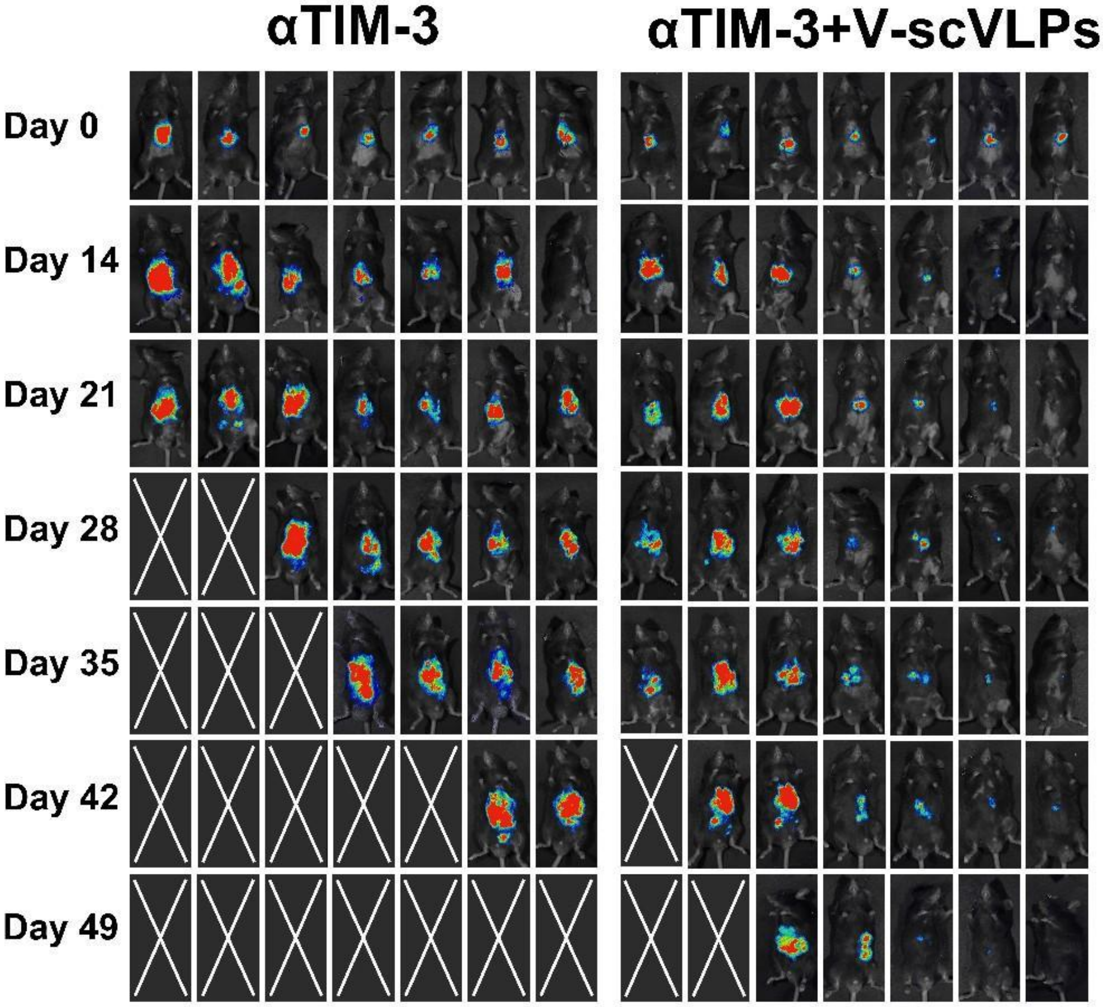

Figure. S25. Bioluminescence imaging of mice at the day 0, 14, 21, 28, 35,42 and 49 before and after receiving different treatments as indicated, n = 7.

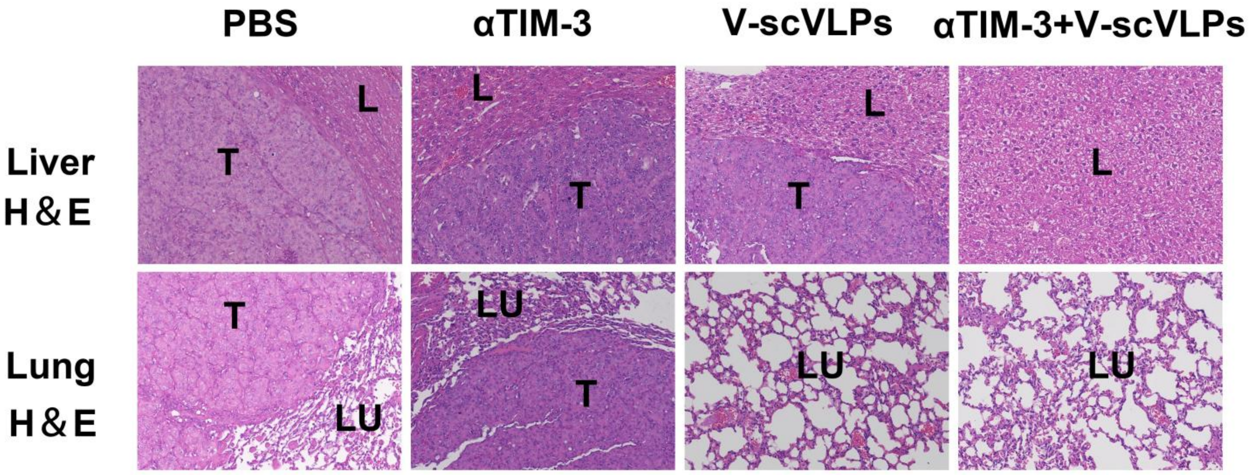

Figure. S26. The H&E staining of liver and lung tissues after receiving different treatments at the 49th day. T represents tumor tissues, L was liver tissues, and Lu was lung tissues.

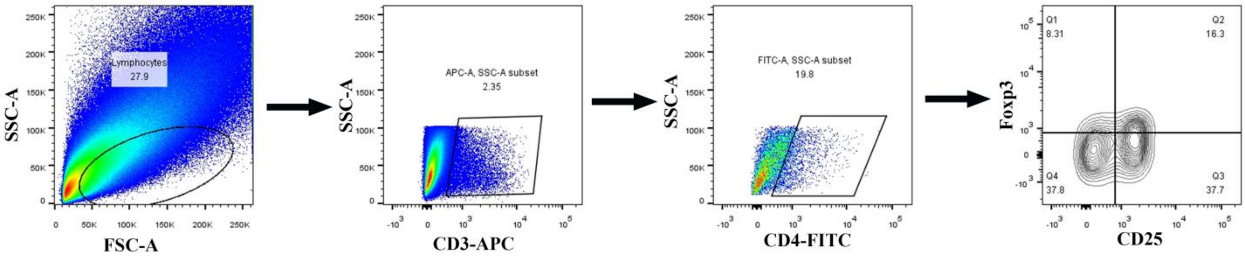

Figure. S27. Identify the different types of $CD3^{+}CD4^{+}CD25^{+}Foxp3^{+}$T cells in tumors after receiving different treatments by FCM with staining anti-CD3-APC, anti-CD8-PE, TIM-3-FITC antibodies.

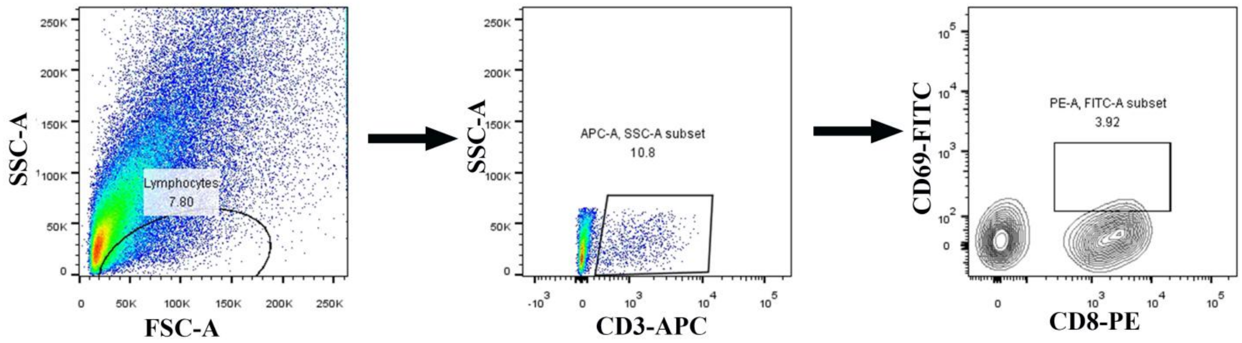

Figure. S28. Identify the different types of CD3$^+$CD8$^+$CD69$^+$T cells in tumors after receiving different treatments by FCM with staining anti-CD3-APC, anti-CD8-PE, CD69-FITC antibodies.

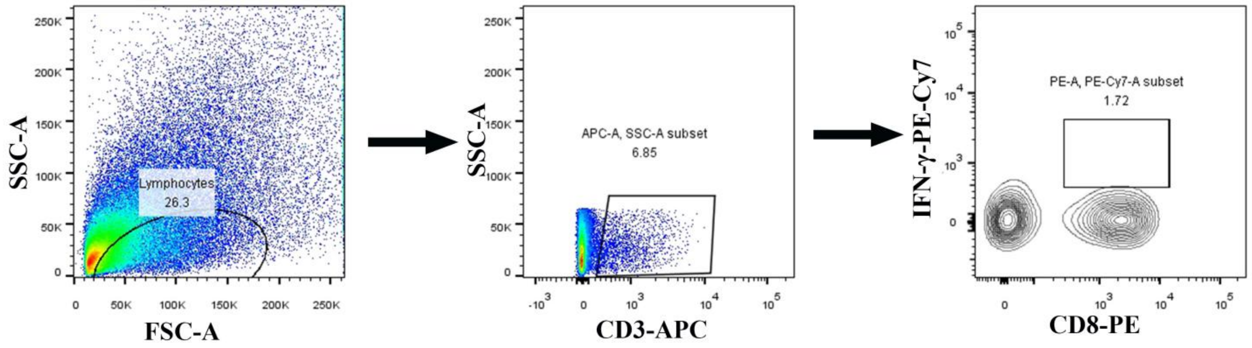

Figure. S29. Identify the different types of CD3$^+$CD8$^+$IFN-γ$^+$T cells in tumors after receiving different treatments by FCM with staining anti-CD3-APC, anti-CD8-PE, IFN-γ-PE-Cy7 antibodies.

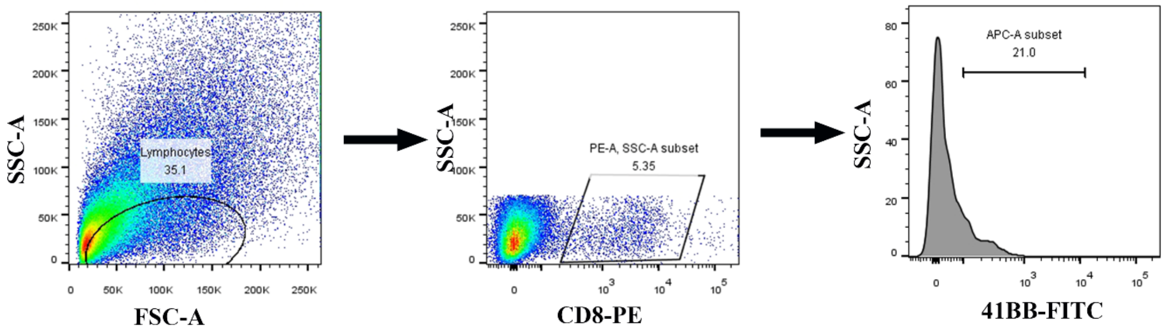

Figure. S30. Identify the different types of CD8$^+$41BB$^+$T cells in tumors after receiving different treatments by FCM with staining anti-CD8-PE, 41BB-FITC antibodies.

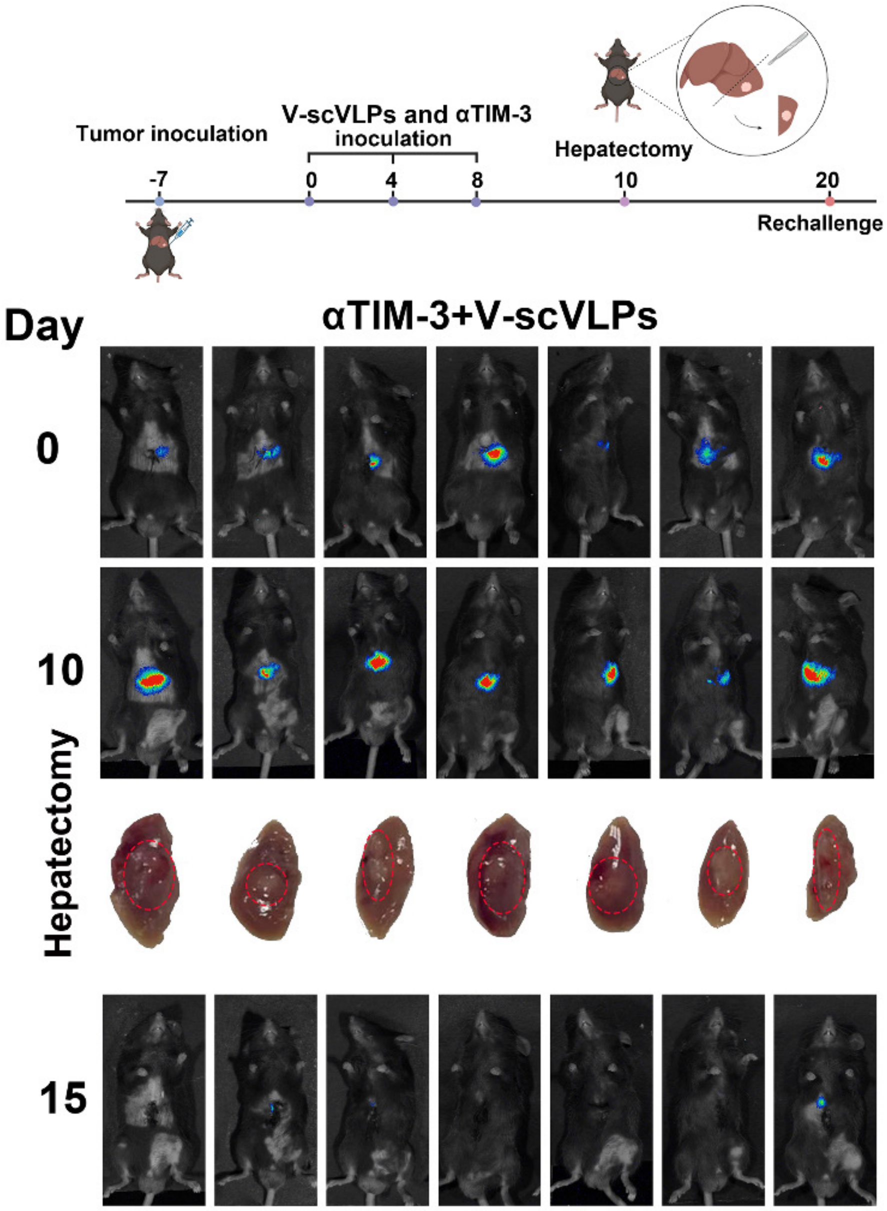

Figure. S31. Bioluminescence imaging of mice at the day 0, 10, 15 and photographs of orthotopic liver tumors and their H&E staining that excised from mice after inoculation Hepa1-6 tumor cells for 10 days, n = 7.

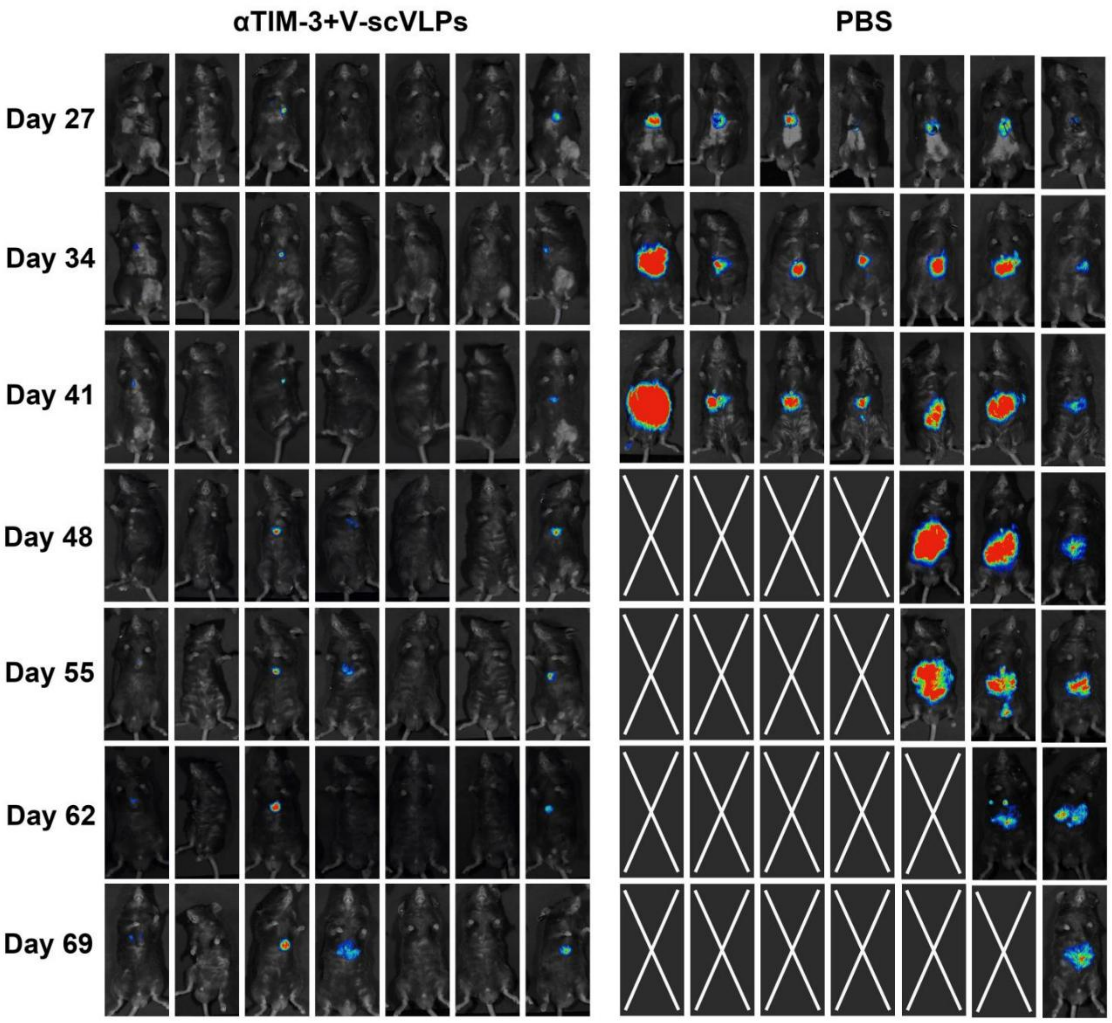

Figure. S32. Bioluminescence imaging of mice at the day 27, 34, 41, 48, 55, 62 and 69 after rechallenge as indicated, n = 7.

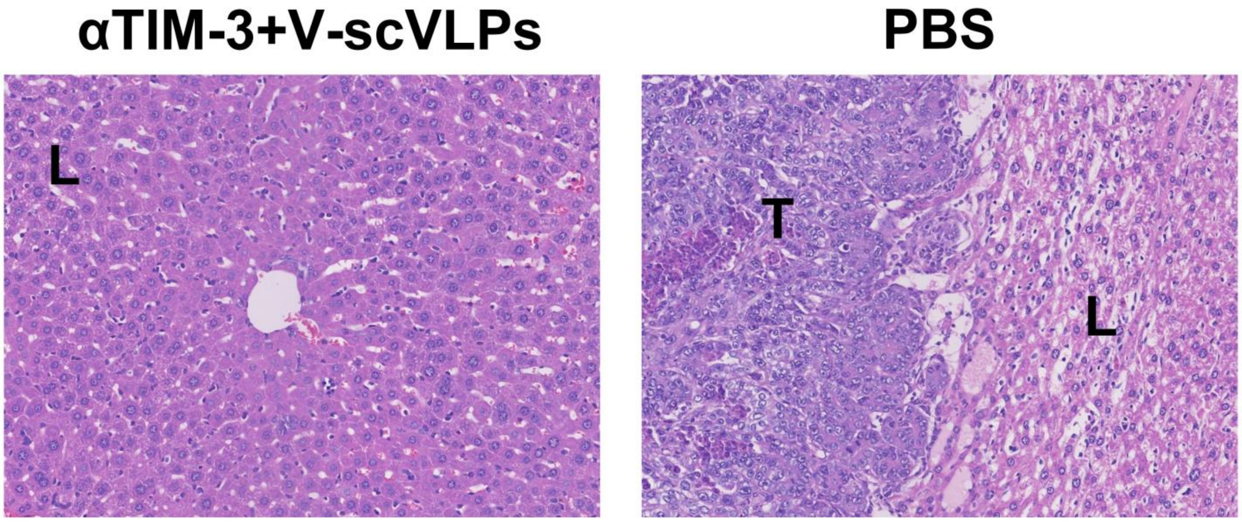

Figure. S33. The H&E staining of liver tissues and tumors after receiving different treatments at the day 69 day. T represents tumor tissues, L was liver tissues.

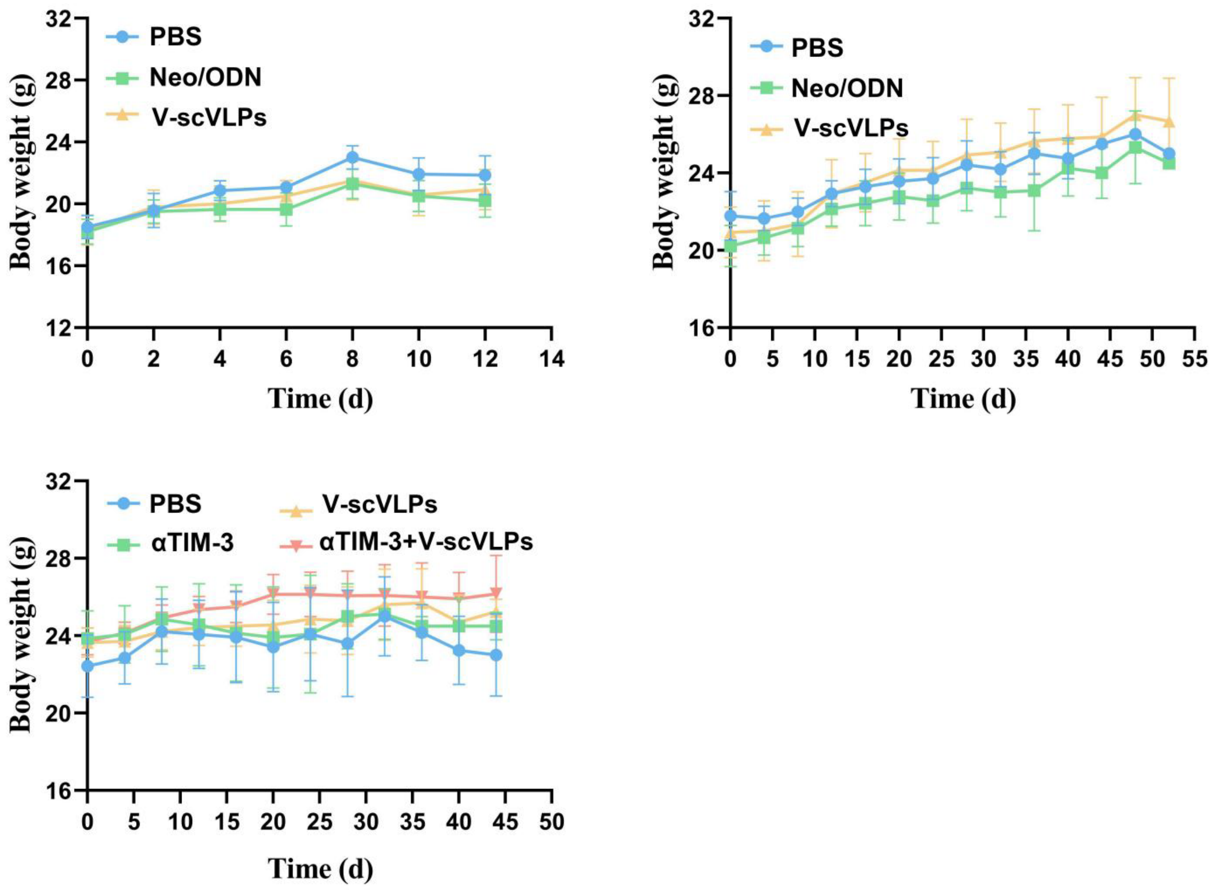

Figure. S34. Weight loss of Hepa1-6 tumor-bearing mice during different treatments as indicated. Data are presented as mean ± SD (n = 7).

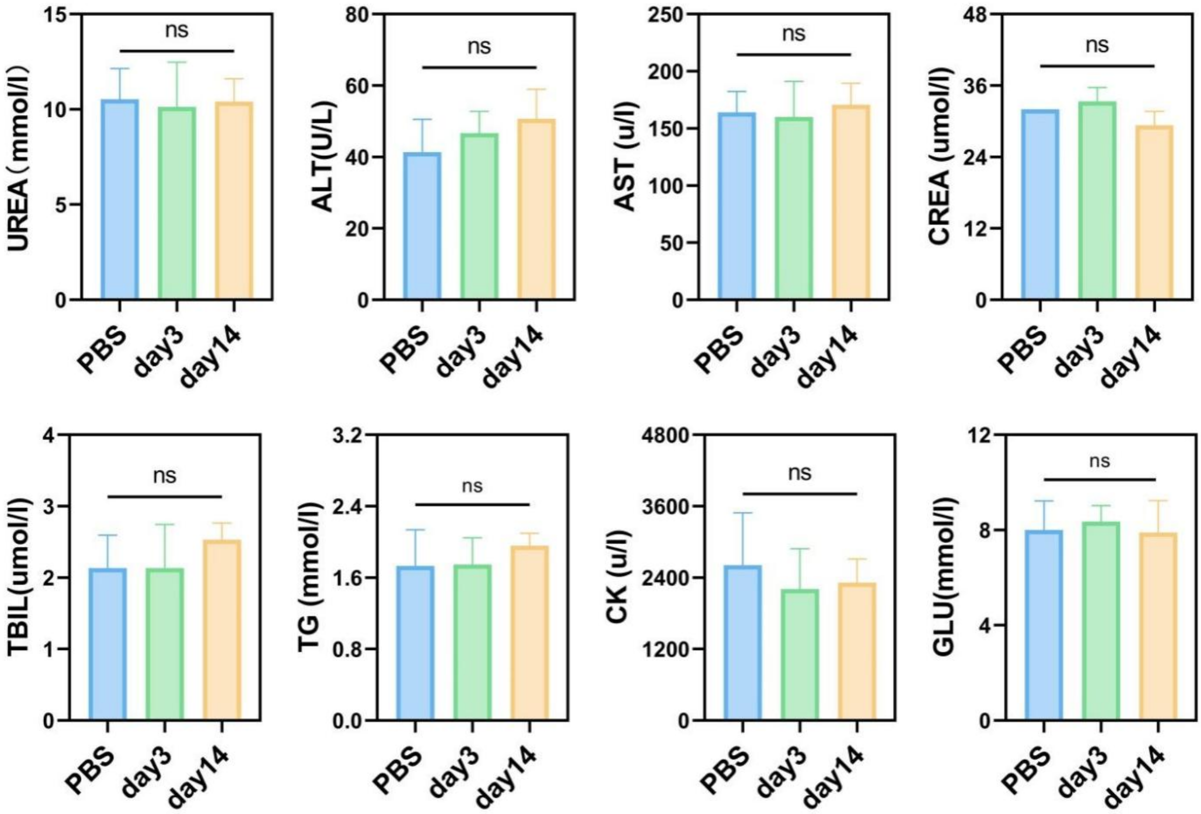

Figure. S35. Biochemical analysis (aspartate aminotransferase, AST; alanine aminotransferase, ALT; creatinine, UREA, Serum urea; CREA; total bilirubin, TBIL; thyroglobulin, TG; Alkaline phosphatase, CK; Glucose, GLU) in healthy mice at 3rd and 14th day after *s.c.* injection of V-scVLPs. Data are presented as mean ± SD (n = 3). The statistical analysis was performed with ANOVA analysis.

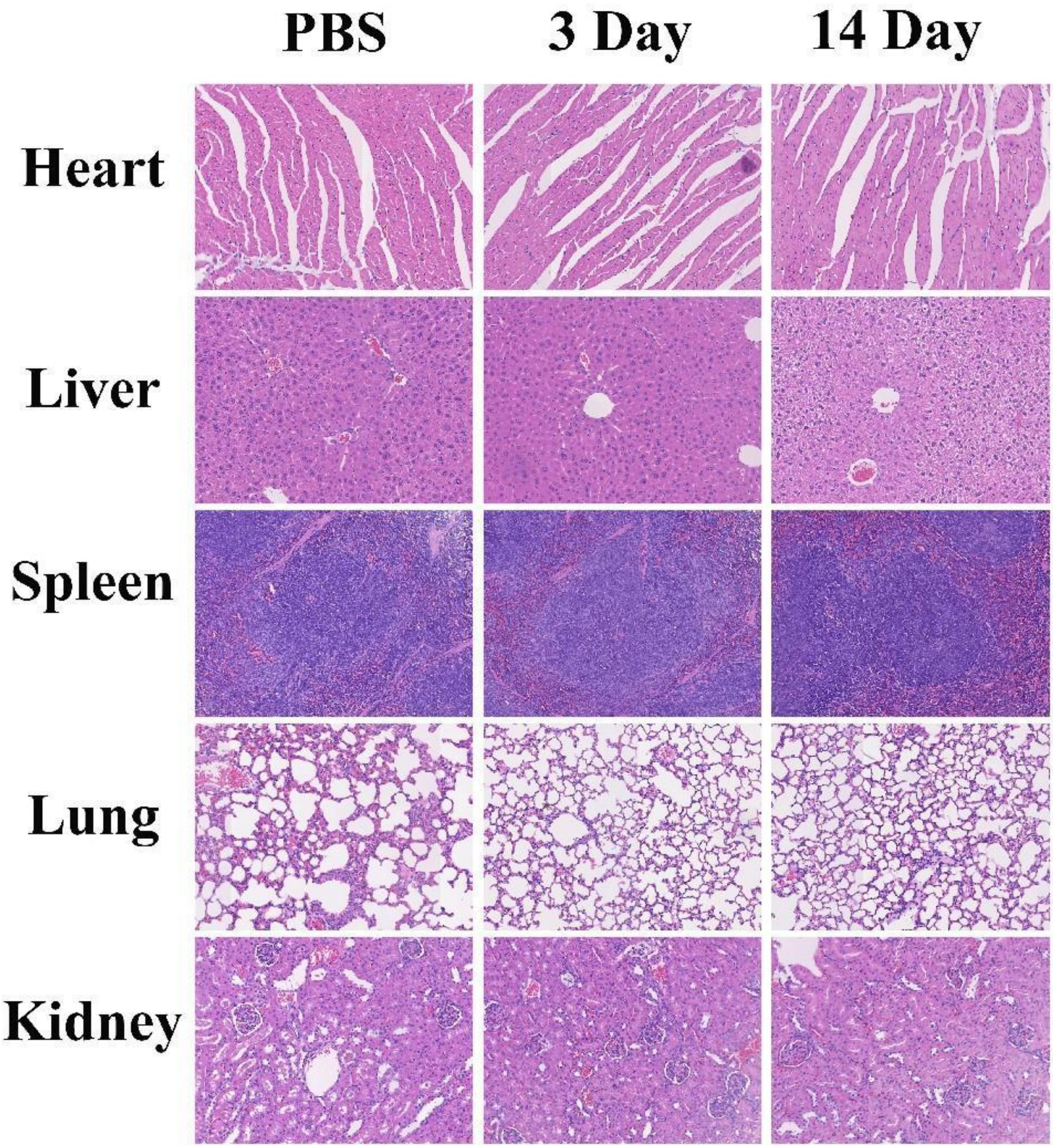

Figure. S36. H&E imaging of major organs (heart, spleen, lung and kidney) from healthy mice after *s.c.* injection with V-scVLPs for 3 or 14 days later.
